# Supplementary material for: Synthetic neural-like computing in microbial consortia for pattern recognition
Source: Nat Commun. 2021 May 25;12:3139. doi: 10.1038/s41467-021-23336-0 (PMC8149857; doi:10.1038/s41467-021-23336-0)
Supplement: Supplementary file 1 — Supplementary Information [file 41467_2021_23336_MOESM1_ESM.pdf]

Supplementary file for

Synthetic neural-like computing in microbial consortia for pattern recognition

Contents

1 Supplementary Notes **2**

1.1 Analog (non-zero) inputs 2

1.2 Repetitions in patterns 2

1.3 Cross-talk discussion 3

1.4 Explanation on classifying 4-bit patterns 4

1.5 More on experiment workflow 5

1.6 Algorithm development 6

1.7 Alternative implementations 9

1.8 Transfer function fitting 10

1.9 Negative weight 10

1.10 Capacity of perceptron and scale-up to multi-layer networks 11

1.11 Fluidics experiments 12

2 Supplementary figures **14**

2.1 Fluorescence histogram figures 36

3 Supplementary videos **43**

4 Supplementary tables **44**

1 Supplementary Notes

1.1 Analog (non-zero) inputs

It is possible to implement a threshold level for input bits using a low analog input with nonzero inducer concentration. As shown from the transfer functions of sender circuits (Fig. 2e), we can select approximately OC6 at 0.05 μM as the threshold level with minimal effects on activating senders such as P_{lux} mut7, mut15 and mut8. For senders with high maximal activities such as mut40, it will be more challenging to implement a nonzero input, due to the higher basal levels.

Furthermore, in our protocol, there is another complication in implementing nonzero inputs in experiments. This complication arises from the fact that we used aTc for "0" input states to turn off the activity of constitutive promoter P_{lacO} on the low-copy-number plasmid, in order to prevent cross-talks when senders exposed to different levels of OC6 are mixed. To implement the nonzero input, when aTc is present, even a high concentration of OC6 would not activate P_{lux} promoters. A low threshold input is thus not applicable to this scenario.

Nevertheless, as a simple demonstration of low "0" inputs, we experimentally examined the circuit performance to a 2-bit pattern set (Supplementary Fig. 1). The inputs were two different chemical inducers, Arabinose and OC6. In this case, circuits for the two inducers are independent. There is little concern for cross-talks and no requirement for aTc depenednt as in Fig. 2a to control cross-talks. A nonzero level of inducers at 1 μM was used for "0" states, and 100 μM for "1" states. As shown in Supplementary Fig. 1, orange bars represent recevier output when no inducer is provided at "0" state and blue bars are for nonzero inducer at "0" state. Compared to the former case, receiver output corresponding to the latter exhibit higher levels at "00" and "01" patterns, but are still capable of separating patterns "00" and "01" versus patterns "10" and "11".

To demonstrate a threshold input without concerning aTc, we simulated the circuit classification results using simulations by varying the inducer concentrations for "0" bits from 0.02 to 0.1 μM and simulated the classification results (Supplementary Fig. 2). As expected, up to 0.05 μM, the classification results stay unaffected. The performance becomes compromised, as the inducer concentration for "0" bits increases.

1.2 Repetitions in patterns

Regarding that only 10 patterns among the 30 are distinct, there are two factors rendering such repetitions. First, three distinct weight values are used for classification. To classify 3×3 patterns in binary values, essentially a few weights are sufficient to provide good classifications (Supplementary Fig. 5). With only a few weight values, many $\vec{p}^T \vec{w}$ products essentially consist of repeating elements. Combining elements by weights allow us to

reduce the size of patterns. Second, these elements can be combined because we directly mix senders during the experiment. In this case, how we mix the senders, that is, the order of elements in $\vec{p}^T\vec{w}$ products does not matter. For example, given a weight vector [1,0,2,1], pattern [1, 0, 1, 0] yields an element-wise product [1,0,2,0]. For pattern [0,0,1,1], the product is [0,0,2,1]. Both products are considered the same in our protocol, because they consist of the same set of elements, albeit in different orders. We included the above explanation in Supplementary Notes section Repetitions in patterns.

1.3 Cross-talk discussion

We performed an assay to show that the cross-talk caused by residual OC6 among senders indeed exists. We designed three 4-bit patterns (in Supplementary Fig. 13) using weights of mut40 and mut8. The "1" states inputs are given to mut8, which is a weak mutated P_{lux} promoter and generates a small amount of OHC14. All bits of the three patterns should correspond to low overall OHC14. Thus, all three patterns should result in low receiver activities. We examined the corresponding receiver output for the three patterns, when senders were pre-incubated with and without aTc (20 $ng\ ml^{-1}$). Without aTc, sender mix lead to higher activation levels in receivers than with aTc.

In our study, both residual OC6 and aTc can affect senders. In the main text, we primarily concern about the cross-talk from OC6 to "0" state senders with P_{lux} activators (positive weight), as discussed in the previous paragraph. In a same way, residual OC6 can also repress "0" state senders with P_{lux} repressors (negative weight). But the repression of OC6 often needs hours to take effect, which is much longer than the 90 min incubation time of sender mix and receivers in experiment (Fig. 3b), and thus is not a concern for our protocol.

Besides, aTc can affect senders. The possible effects of aTc are illustrated in Supplementary Fig. 12a for explanation. Senders with P_{lux} activators (the orange line) and P_{lux} repressors (the blue line) are incubated with OC6 first and then diluted in exposure to aTc. Hypothetically, for positive weights senders at "1" state, exposure to aTc tends to reduce promoter activity, due to a decreasing activity of P_{lacO}. This process would result in a slowed production of OHC14. However, this effect would not significantly decrease the amount of OHC14, because lactones are highly stable, estimated based on the slow degradation rate of OC6 [1].

For senders containing P_{lux} repressors, turning off P_{lacO} by aTc tends to reactivate repressor promoters. Nonetheless, the residual aTc at 0.2 $ng\ ml^{-1}$ does not reactivate P_{lux} repressors until approximately two hours. These temporal dynamics are shown in Supplementary Fig. 12b by time course data collected from a plate reader. In the assay, we incubated P_{lux} repressor senders in OC6 (100 μM) for four hours. Senders were subsequently diluted 100 times and incubated in aTc with varying concentrations. The

OD600 and mCherry levels were monitored across time at every 15 min. We noticed an increase in mCherry level around 150 min. In our protocol, the sender mix was incubated with receivers for 90 min, which is within the safe period of two hours.

To experimentally demonstrate that aTc will not reactivate P_{lux} repressor senders, we carried out an assay by mixing P_{lux} activator senders incubated with aTc (senders at "0" state) and P_{lux} repressor senders incubated with OC6 (senders at "1"). In the three 4-bit patterns (Supplementary Fig. 12c), all bits are expected to generate low OHC14. Thus, unless P_{lux} repressors become reactivated, all patterns should result in low signal output in receivers. After incubation, we examined whether receivers produce EYFP due to the possible reactivation of P_{lux} repressor senders. As expected, no activation of receivers was observed (Supplementary Fig. 12c).

1.4 Explanation on classifying 4-bit patterns

Hypothetically, we can adjust the receiver transfer function to change the boundary range and improve pattern classification. As a demonstration, we simulated the classification performance using a transfer function with an increased hill-coefficient n following the equation as below.

$$f(x) = \beta_m \frac{(x/K_d)^n}{1 + (x/K_d)^n} + \beta_0 \beta_m$$

Here K depends on the binding affinity between the promoter and inducer-transcription factor (TF) complex. n describes the cooperativity of binding. As shown in Supplementary Fig. 16, increasing n from 2.33 to 4.0 leads to a sharpened receiver transfer function (Supplementary Fig. 16a). Here the parameter n that equals 2.33 is obtained by fitting experimentally measured mCherry levels in senders and the corresponding EYFP levels in receivers (Fig. 4c). With n equals 4, we improved classification results (Supplementary Fig. 16b), as the four patterns within the boundary zone (grey bars) become more separate from the patterns in the "high" category (six blue bars on the right).

Notably, n equals 4 is much higher than the cooperativity involved in synthetic biomolecular interactions. Nevertheless, one possible way to achieve ultra-sensitivity is to cascade multiple layers of genetic circuits [2]. We demonstrated this mechanism by simulating the transfer function of a two-layer genetic circuit (Supplementary Fig. 16c). The first layer contains a promoter that drives a gene to produce TF that activates the promoter on the second layer. In both layers, n and K are kept unchanged as 2.33 and 2820 respectively and both values are biologically realistic. This combination of parameters results in an overall transfer function that approximates the target function with the desired n value of 4.

For the arbitrarily selected weight set [450, 3500, 900, 3500], the products of weight and 16 patterns are [0, 3500, 900, 3500, 450, 1350, *4400, 4400, 3950, 3950*, 7000, 7900,

4850, 7450, 4850, 8350]. Values for the four patterns within the boundary are italicized and underlined. When n equals 2.33, the corresponding network output calculated as in Fig. 4a is [0.02, 0.39, 0.04, 0.39, 0.02, 0.08, *0.52, 0.52, 0.46, 0.46*, 0.77, 0.82, 0.57, 0.79, 0.57, 0.84]. In this case, [4000, 4500] that results in output values [0.46, 0.53] was intuitively selected as a boundary range to partition the 16 patterns into two equal sets. With n equals 4, the network output becomes [0.02, 0.3, 0.02, 0.3, 0.02, 0.03, *0.52, 0.52, 0.41, 0.41*, 0.88, 0.93, 0.61, 0.91, 0.61, 0.95]. The same output values [0.46, 0.53] corresponds to [4150, 4450] in sender fluorescence, which is narrower than [4000,4500]

We carried out an experiment for all 16 patterns as shown in Supplementary Fig. 16d. Six patterns grouped on the left of the graph correspond to pattern set0 in Fig. 3a. Another six patterns grouped on the right of the graph correspond to pattern set1. Four patterns labeled in grey color placed in the middle of the graph correspond to the additional four patterns that are not included for classification. As shown in Fig. 3a, we used weight values [450, 3500, 900, 3500] and calculated summation of mCherry values for the 16 patterns. We picked a lower bound 4000 and a higher bound 4500 to separate patterns into distinct categories. In Fig. 3a, the corresponding summed mCherry levels are listed beside each pattern. The overall mCherry levels for the additional four patterns equal 4400, 4400, 3950, and 3950 respectively, which overlap with the region between two bound values and thus are not easy to classify.

1.5 More on experiment workflow

Regarding the workflow, three duration parameters are particularly relevant. First is the sender incubation period. We expect it to be long enough for cell growth so that activator senders can reach proper activities and levels of repressor senders can be sufficiently reduced. Meanwhile, the incubation should not be excessively long such that over amount of cells may result in inducer being effectively diluted and losing effects, especially for inducers of TF repressors. We measured the cell growth in OD600 across time using a plate reader (Supplementary Fig. 17). Based on the slopes of growth curves, the fast growth period ends between 200 to 300 min.

After diluting incubated senders 100 times, we allow senders to incubate for another 30 min, which is approximately one cell cycle. This step serves as a slight adjustment to OD after dilution so that OD levels can reach approximately 0.1, within the measurable range of the plate reader. All sender solutions should be of roughly equal OD levels.

The third duration parameter is the incubation time between sender mix and receivers. As mentioned in section Cross-talk discussion, a safe period of two hours has been considered to prevent inactivated P_{lux} repressor promoters become reactivated. Our protocol using 90 min is within a safe time. To ensure sufficient cell densities for flow

cytometry measurement, receivers were initially diluted 50 times from overnight culture. The volume ratio of sender to the receiver was selected after trials. These numbers can be adjusted depending on cell growth conditions.

1.6 Algorithm development

Consider an N-bit input pattern $\vec{x}_j$, where j is an index from 1 to M, representing different input patterns. The ith element of the input vector is $\vec{x}_{ji}$, corresponding to a single OC6 level for pattern j. The algorithm thus aims to find an optimal weight vector $\vec{w}$ with N elements. For each pair of x_{ji} and w_i, we define a basis function $\phi_{ji}(x_{ji}, w_i)$, which represents a transformation of chemical input to senders depending on whether the sender is activated (P_{lux} activator) or repressed (P_{lux} repressor) by OC6. Thus, the total activity of P_{lux}, as well as the total amount of OHC14 being produced (measured in mCherry level), can be described using the product of weight and input vector as follows

$$\vec{w}^T \cdot \vec{\phi_j}(\vec{x_j}, \vec{w}) = \sum_i w_i \phi_{ji}(x_{ji}, w_i) \tag{1}$$

To describe $\phi_{ji}(x_{ji}, w_i)$ using experimentally measured parameters (Fig. 2e, Table 1), we first define $\phi_{pos}(x_{ji})$ and $\phi_{neg}(x_{ji})$, and then decompose $\phi_{ji}(x_{ji}, w_i)$ in terms of these two expressions. $\phi_{pos}(x_{ji})$ and $\phi_{neg}(x_{ji})$ are in the form of Hill equation as follows

$$\phi_{neg}(x_{ji}) = \frac{1}{1 + (x_{ji}/K_{d1})^{n_1}} + \alpha_0 \tag{2}$$

$$\phi_{pos}(x_{ji}) = \frac{(x_{ji}/K_{d2})^{n_2}}{1 + (x_{ji}/K_{d_2})^{n_2}} + \beta_0 \tag{3}$$

where in eq2, K_{d1} (0.252) describes the dissociation constant between TF binding and the P_{lux} repressor promoter. n_1 (0.669) is the Hill coefficient, indicating binding cooperativity of TF and P_{lux}. Similar definitions apply to parameters in eq3, in which K_{d2}=50, β_0=0.1, n_2=0.45.

Notably, although parameters such as K_{d2}, n_2, and β_0 take different values in Table 1, they are set as constant variables in eq3. This is to simplify the transfer functions of mutated P_{lux} by reducing variables. From the fittings in Fig. 2d and sensitivity analysis (Supplementary Fig. 14), we can tell that the transfer functions are mostly affected by β_m (i.e. the maximum promoter activity) and less sensitive to other parameters. Hence, we factor out the dominant parameter β_m and consider it as the weight value. In this case, the maximum activity in eq3 is uniformly $1 + \beta_0$, and the product equals the complete fitted transfer function, and represents the activity of a mutated P_{lux} activator promoter.

Given $\phi_{pos}(x_{ji})$ and $\phi_{neg}(x_{ji})$, $\phi_{ji}(x_{ji}, w_i)$ can be written as

$$\begin{aligned}\phi(x_{ji}, w_i) &= \frac{1}{2}(1 + \frac{w_i}{|w_i|})\phi_{pos}(x_{ji}) + \frac{1}{2}(1 - \frac{w_i}{|w_i|})\phi_{neg}(x_{ji}) \\ &= \begin{cases} \phi_{pos}(x_{ji}), & w_i > 0 \\ \phi_{neg}(x_{ji}), & w_i < 0 \qquad i = 0, ..., N-1 \end{cases}\end{aligned} \tag{4}$$

where the expressions $\frac{1}{2}(1 + \frac{w_i}{|w_i|})$ and $\frac{1}{2}(1 - \frac{w_i}{|w_i|})$ are signs of w_i. For positive weights, the second term disappears. Likewise, the first term disappears for negative weights. We consider ϕ_{pos} and ϕ_{neg} as the unit transfer functions. It then follows that the total production of signaling molecules OHC14, as described using the weight vectors and basis functions, can be formulated as follows

$$\begin{aligned}\vec{w}^T \vec{\phi}_j(\vec{x_j}, \vec{w}) &= \sum_{i=0}^{N-1} \frac{w_i}{2}(1 + \frac{w_i}{|w_i|})\phi_{pos}(x_{ji}) + \frac{w_i}{2w_i}(1 - \frac{w_i}{|w_i|})\phi_{neg}(x_{ji}) \\ &= \sum_{w_i>0} w_i \phi_{pos}(x_{ji}) + \sum_{w_i<0} \phi_{neg}(x_{ji})\end{aligned} \tag{5}$$

Accordingly, for positive weights, w_i corresponds to various β_m values. And for negative weights, $w_i$corresponds to various α_m values.

When mixing senders and receivers, the product in eq5 serves as the input to activate receivers. We experimentally measured the activation function σ, by mixing senders that have been incubated in various OC6 levels, with receivers in a 1-to-19 volume ratio. Then σ is fitted using a Hill equation (Fig. 4c), similar to the fittings in Fig. 2d, but with mCherry levels in senders as the horizontal axis. Accordingly, for a pattern $\vec{x}_j$, the output of receivers is

$$R_j = \sigma(\frac{1}{N}\vec{w}^T \vec{\phi}_j(\vec{x_j}, \vec{w})) \qquad j = 0, ..., M-1 \tag{6}$$

For each pattern $\vec{x}_j$, we define a target vector $\vec{t_j}$, which consists of elements either equal to a low target value (t_{low}, e.g., 0) or a high target value (t_{high}, e.g. a value similar to β_m as the fitting in (Fig. 4c). Target vectors are assigned with the same values for patterns belong to the same category. To search for proper weights, we adopted a sequential learning algorithm based on gradient descent [3], with the error gradient defined as

$$\nabla \vec{E} = \sum_{j=0}^{N} min((R_j - t_j), t_{high}) \vec{\phi}(\vec{x_j}) \tag{7}$$

$$\vec{w}^{\tau+1} = \vec{w}^{\tau} - \eta \nabla \vec{E} \tag{8}$$

where τ refers to iteration number. The weight values are updated at every iteration by the amount $\eta\nabla\vec{E}$, where η is the learning rate to adjust the speed of learning, the same as the step size shown in Fig. 4b. We choose η a value so that the output $\vec{R}$ approaches a steady state within 300 iterations. In particular, we formulated eq7 in analogous to equation (3.12) in [**Bishop**], which was developed using logistic activation functions.

We used a metric to quantify the performance of classification. For a $\vec{w}$, we can calculate a product vector using the corresponding output $\vec{R}$ as follows

$$\vec{p} = (2\vec{t} - \vec{t_H})(2\vec{R} - \vec{t_H}) \tag{9}$$

where the target vector $\vec{t}$ contains elements that are either t_{high} or t_{low} and all elements in $\vec{t_H}$ are t_{high}. Hence, the first term $2\vec{t} - \vec{t_H}$ yields a vector consists of either $-t_{high}$ or t_{high}. In the second term, the elements in $\vec{R}$ are within the range from 0 to t_{high}. Thus, elements in $2\vec{R} - \vec{t_H}$ also vary from $-t_{high}$ to t_{high}. In the optimal case, $\vec{R}$ approaches $\vec{t}$ and $2\vec{R} - \vec{t_H}$ approaches $2\vec{t} - \vec{t_H}$. Accordingly, all elements in $\vec{p}$ approach $(-t_{high})^2$ or t_{high}^2, both equal to t_{high}^2. To summarize the vector into a single scalar, intuitively we define the metric

$$product\ measure = (t_{high}^2 - Mean(\vec{p})) + 10 \cdot Var(\vec{p}) \tag{10}$$

For the optimal solution, $\vec{p}$ in eq9 is expected to contain elements that uniformly approximate t_{high}^2. Therefore $\vec{p}$ has a mean value close to but always less than t_{high}^2, and exhibits a small dispersion. Accordingly, a good classifier is expected to generate a small product measurement. The coefficient 10 in eq10 is chosen arbitrarily to match the scale between mean value and variance.

The perforamce of gradient-based method relies on parameters such as η and may not always yield optimal weights for classification. In this case, we adopted an optional step two algorithm to search for weights. Using the gradient-based method, we obtained an initial weight vector. Then we used a direct method called Nelder-Mead simplex method [4] to obtain the optimal solution that minimize the product measure as defined in eq10.

We used the formulas presented above to update weights for classification of 3×3 patterns. We started with a scenario that positive weight $beta_m$ can be any unbounded non-zero value, whereas negative weight $\hat{\alpha_m}$ can take only one value as in the measured P_{lux} repressor promoter. That is, they can take any positive or negative numbers (Supplementary Fig. 3a). The error gradient $\nabla\vec{E}$ is calculated as the derivative of $\vec{E}$ with respect to $\vec{w}$. In this scenario, positive weights update can take continuous steps. In experiments, we constructed only one type of repressor and four mutated P_{lux} activator promoters with distinct maximum activities (Table1). Thus for a more realistic scenario, we constrained the algorithm by allowing weights take only discrete levels. We adapted

the gradient-based algorithm to handle the case of discrete weight values. Inspired by the algorithm in [5], we introduced a hidden variable $\vec{h}$ that can be continuously updated as in eq8 (Supplementary Fig. 4). Then $\vec{w}$ was adjutsted as a function of $\vec{h}$. We pre-selected a set of weight values. Each element of $\vec{w}$ can be updated only when the corresponding element in h_i hits one of pre-selected values, depending on the sign of $\nabla\vec{E}$.

Using the algorithm, we updated weights to classify 3×3 patterns assuming positive weights take continuous unbounded non-zero values (Fig. 5b) or discrete values. We calculated the product measurement (eq10) across iterations. As expected, this metric decreased smoothly for continuous weights and adjusted in steps for discrete weights (Supplementary Fig. 6a). Using the minimal values of product measurement as an indicator for classification performance, we demonstrated that discrete weight values perform as well as as continuous weight values (Supplementary Fig. 6b). Even a minimal weight set that contains two levels suffices to classify 3×3 patterns (Supplementary Fig. 5).

Does it suggest that any two distinct weight levels are sufficient for classification? Considering that genetic circuits are highly noisy, we hypothesize that a certain size of weight set is essential to provide the redundancy to tolerate noise from genetic circuits. To examine how the number of weight levels affects classification performance in case of noisy weights, we evaluated the minimal values of product measurement when weight set contains 5, 3 and 2 distinct levels (Supplementary Fig. 6c). As expected, as weight levels increase, the minimal product measurement exhibts a decreasing mean value with reduced variance (Supplementary Fig. 6b).

1.7 Alternative implementations

We also explored several alternative implementations of perceptrons based on intercellular communications using fluidic devices, modified regulation circuits in senders and network for classifying non-spatial patterns. These implementations do not rely on using the aTc dependent double inversion circuit to reduce cross-talks.

Microfluidics are widely used in synthetic biology studies to compartmentalize reactions. To keep sender reactions spatially separated, we built a three-well device as in Supplementary Fig. 7 with senders in the side wells and receivers in the center well. In this case, senders for individual pixels are separated and located apart. Signaling molecules OHC14 can diffuse across channels to reach the center well. We carried out the experiment without heavy manual operations. Experimental details are provided in the section Fluidics experiments.

In another implementation, we implemented sender circuits with positive feedback (PF) regulation (Supplementary Fig. 10a) by replacing the P_{lacO} promoter with a mutated P_{lux} that matches with the promoter driving the *cinI* and *mCherry* genes. The transfer functions of PF circuits are not as linear as those for senders with open loop (OL) circuits

(Fig. 2e). Nevertheless, the PF circuits exhibit higher activation thresholds than OL circuits. This leads to a desirable property that cross-talks between senders can be managed by merely diluting the senders 100 times, and the residual inducer concentration can be reduced below the threshold. We verified this property using an assay of 4-bit patterns as shown in Supplementary Fig. 10b. Furthermore, despite their nonlinear transfer functions, the PF sender circuits can classify 4-bit patterns (Supplementary Fig. 11). In this case, due to the steep transfer functions, PF circuits are sensitive to inducer concentrations. Thus, a proper inducer concentration for "1" states needs to be chosen carefully to keep senders in the correct activation level.

Furthermore, it is possible to interpret each bit or pixel as a distinct chemical inducer, and perform a "one-pot" experiment using circuits constructed to sense these chemical inputs (Supplementary Fig. 1). As a demonstration, we constructed a circuit to sense Arabinose, using the same topology as the circuit for OC6. Two types of sender circuits were mixed with or without individual inducers, and incubated with receivers for 3 hours in the same reaction compartment. Afterwards, we examined the activities of receivers, showing that receivers were activated to distinct levels, in response to combinations of sender patterns.

1.8 Transfer function fitting

We fitted all transfer functions of genetic circuits in the study using Hill equations. The median levels of measured fluorescence data were averaged for fitting. As described in the main text, activation functions were fitted using equation in the form $f(x) = \beta_m \frac{(x/K_d)^n}{1+(x/K_d)^n} + \beta_m \beta_0$, whereas the repression functions was in the form $f(x) = \alpha_m \frac{1}{1+(x/K_d)^n} + \alpha_0 \alpha_m = \hat{\alpha}_m(\frac{(x/K_d)^n}{1+(x/K_d)^n} - \alpha_0 - 1)$. In particular, β_m has positive values, representing the upward slope in transfer functions; α_m has a negative value, indicating the downward slope of the transfer function. Fittings were carried out using a built-in function curve_fitting from Python Scipy package.

We also performed a sensitivity analysis for fittings of P_{lux} activator transfer functions. We used a variance based global sensitivity analysis [6] from a Python package SALib [7] (v1.3.12). The analysis result indicates that the transfer functions are most sensitive to β_m (Supplementary Fig. 14). Therefore, we simplified the model for P_{lux} activator transfer functions by factoring out the β_m and setting other variables as constant.

1.9 Negative weight

In general, we considered two possible approaches for implementing negative weights. The first approach is using a repressor promoter, as we implemented in the study. In the absence of inducers, the promoter activity is high. Thus, a summation of the negative weight and positive weight promoters results in an amount of OHC14 in an approxi-

mate form $w_1x_1 + (const - w_2x_2)$, where w_1 and w_2 are positive and negative weights, respectively. This implementation leads to an approximate linear summation of OHC14.

A second approach involves degradation proteins. OHC14 can be inactivated by quorum quenching (QQ) enzymes [8], which include lactonases, acylases, oxidoreductases and other enzymes. Thus, a possible solution to decrease the amount of OHC14 is to express these QQ enzymes. However, this approach has two drawbacks. First, the enzyme is not specific to OHC14 but can degrade a range of lactones, including the input inducer OC6. Second, unlike lactones, the enzyme cannot diffuse to outsize of cells. This adds to complexities of implementation to achieve linear summation of OHC14.

We adopt the first approach for implementation. In this approach, positive weight refers to the maximum promoter strength variable β_m in a fitted transfer function of an activator promoter, which has a positive value and describes the upward slope of the transfer function. For a repressor promoter, the corresponding weight variable is $\hat{\alpha}_m$, which has a negative value. Intuitively, a negative weight describes the downward slope of the repressor promoter transfer function. A repressor promoter shows high promoter activity in the absence of the inducer and low promoter activity in the presence of the inducer. Thus, when mixing senders of positive and negative weights, whether or not a negative weight decreases the overall amount of OHC14 depends on the input patterns.

To demonstrate experimentally, we examined a set of 2-bit patterns using a negative weight and a mutated positive weight. The two senders were incubated separately with or without inducers and mixed according to four pattern combinations (Supplementary Fig. 15). The mCherry levels of sender mix solutions were measured using flow cytometry. Among the four patterns, pattern "10" (OC6 for positive weight sender only) results in the highest overall mCherry, whereas pattern "01" (OC6 for negative weight sender only) results in the lowest overall mCherry. Thus, the effect of negative weights depends on inducer patterns. Their presence can add to the overall promoter activities when they are not exposed to OC6.

1.10 Capacity of perceptron and scale-up to multi-layer networks

Mathematically, there is an upper limit of recognizable patterns for a $n \times n$ bits array. A perceptron can recognize only linearly separable patterns. A function counting theorem [9] states that for p random patterns and N synapses (or weights, here $N = n^2$ for a $n \times n$ bits array), the total number of arbitrary binary labels on these patterns that allow linear separability is $C(p, N) = 2\sum_{i=0}^{N-1}\binom{p-1}{i}$. As N increases, $C(p, N)$ increases as well, but the ratio of this number to the total possible binary labels 2^p drops quickly after p becomes larger than $2N$. When p equals $2N$, half of the binary labels are linearly separable and can be realized using perceptrons. Thus for large N, the value 2 bits per

synapse has been considered as the capacity of a perceptron with N synapses [9].

The storage capacity of correlated patterns, as in our case for patterns within the same class, is generally higher [10]. When patterns and weights are restricted to binary values, the capacity decreases and were estimated with a maximum of 0.83 bit per synapse [11]. The sparseness of patterns also affect capacity. In particular, capacity is improved for patterns with high sparseness [5].

Since the limited number of patterns discussed above is inherent to perceptron, constructing multi-layer networks can overcome the limitation of linear separability and expand the repertoire of recognizable patterns. Increasing the network layers certainly adds the complexity of the architecture to classify more sophisticated patterns. This has been shown by [12] that a second layer cascade allows the genetic circuit to compute XOR. However, multi-layer networks also require signaling molecules that are mutually orthogonal to reduce cross-talks between network layers. A recent work by [13] on a library of quorum sensing lactone devices would be helpful to design and construct multi-layer networks. Moreover, the transfer function of each layer requires adjustment to optimize the dynamic range. Fine tuning each layer can be realized by mutating relevant promoters and modifying the circuit topology via regulations.

1.11 Fluidics experiments

The fluidic devices were fabricated using a mixed solution that consists of 90% polydimethylsiloxane (PDMS) and 10% cross-linking reagent. The solution was injected using a syringe (approximately 7 ml) into a mold that was built from a petri dish (35 mm in diameter). In the center of the petri dish, an adhesive foil strip (length×width×height=26 mm×5 mm×100 μm) was sticked onto the dish. Subsequently, we placed the devices in vacuum chamber for PDMS degasing. The devices were then placed in a dissipator chamber overnight. On the following day, we took out the hardened PDMS from the petri dish mold. Fluid channels were naturally formed once the PDMS peeled off from the foil strip. For each device, we punched holes along the fluid channel using a biopsy punch (6 mm diameter). Afterwards, devices were sealed on glass slides using plasma activation and placed in the 60 °C incubator overnight.

We used a microscope to observe time-lapse movement of bacteria within a device from one side well to the center. We filled the device with LB media that contains appropriate antibiotics. After a short degasing step to remove the air bubbles in the channel, we drew out the media from wells and refilled wells with 100 μl media using a multi-channel pipette. This step guarantees that solution is simultaneously filled in wells. Then we added 1 μl solution from overnight grown cell culture to a side well and placed a transparent lid over the device to minimize fluid evaporation during incubation. The device was then placed onto a sample holder of the microscope (Nikon T2) for time-lapse

recording. The microscope was encased in a chamber, the temperature of which was adjusted to 37 °C. We used the software Nikon elements to control the recording so that the camera (Andor neo) captured the field of view (FOV) at every 10 min with a 20× magnification. We started recording after bacteria could be identified with clear a focus. Recordings were at a fixed FOV for approximately 3 hours and then the focus view was moved either leftward or rightward to track the movement of bacteria population. We processed the recording file using ImageJ software to align the images.

For the experiments regarding classifying 2-bit patterns, we tested the patterns (in the order 11, 00, 10, and 10) in three fabricated devices. We filled LB media in channels, drew out the residual solution in wells and refilled the wells with 100 μl LB media again using a multi-channel pipette. We added 1 μl recever cell solution from overnight culture to well2. We then mixed equal volumes of sender cell solution and OC6 from 1 mM stock, and added 2 μl from the mixed solution to well1 and well3. The devices were covered with transparent lids and placed in incubator at 37 °C for four hours. Afterwards, we used a multi-channel pipette to draw from the three wells on each device into a 96-well plate and measured the fluorescence levels using FACS. Devices were rinsed with flowing water and autoclaved for the next round of test.

2 Supplementary figures

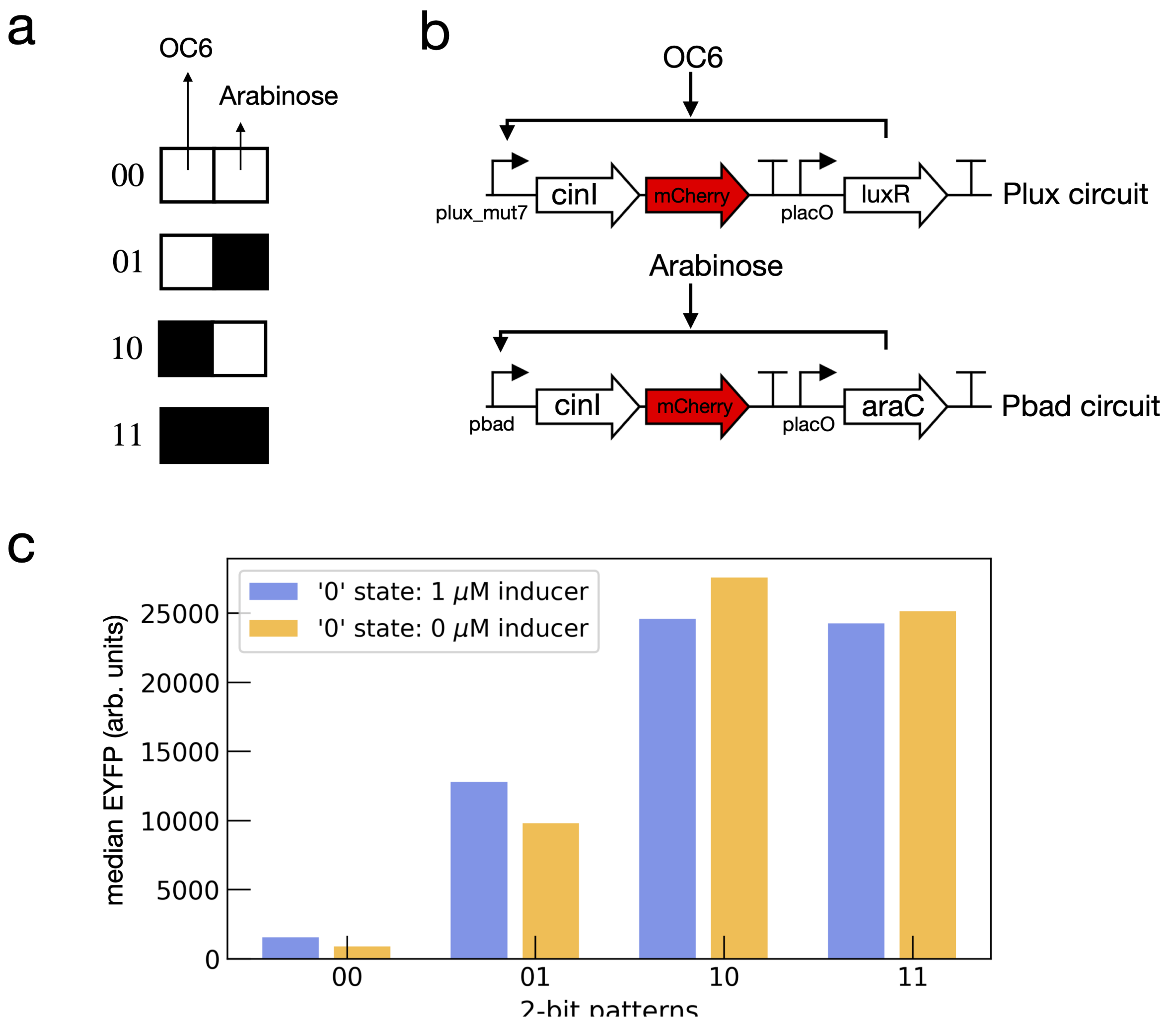

Supplementary Fig. 1: **"one-pot" reaction experiments.** **a** 2-bit patterns. The first bit represents the concentration of inducer OC6 in a binary form, either high ("1", 100 μM) or low ("0", 0 μM or 1 μM). The second bit represents the concentration of inducer Arabinose, also 100 μM for "1" state and 0 or 1 μM for "0" state. **b** Genetic circuits to sense OC6 (P_{lux} circuit with a mutated promoter) or Arabinose (P_{bad} circuit). **c** Median EYFP levels from receivers for the four patterns when "0" state contains no inducers (orange bars) and 1 μM inducers (blue bars). All reactions are incubated for three hours.

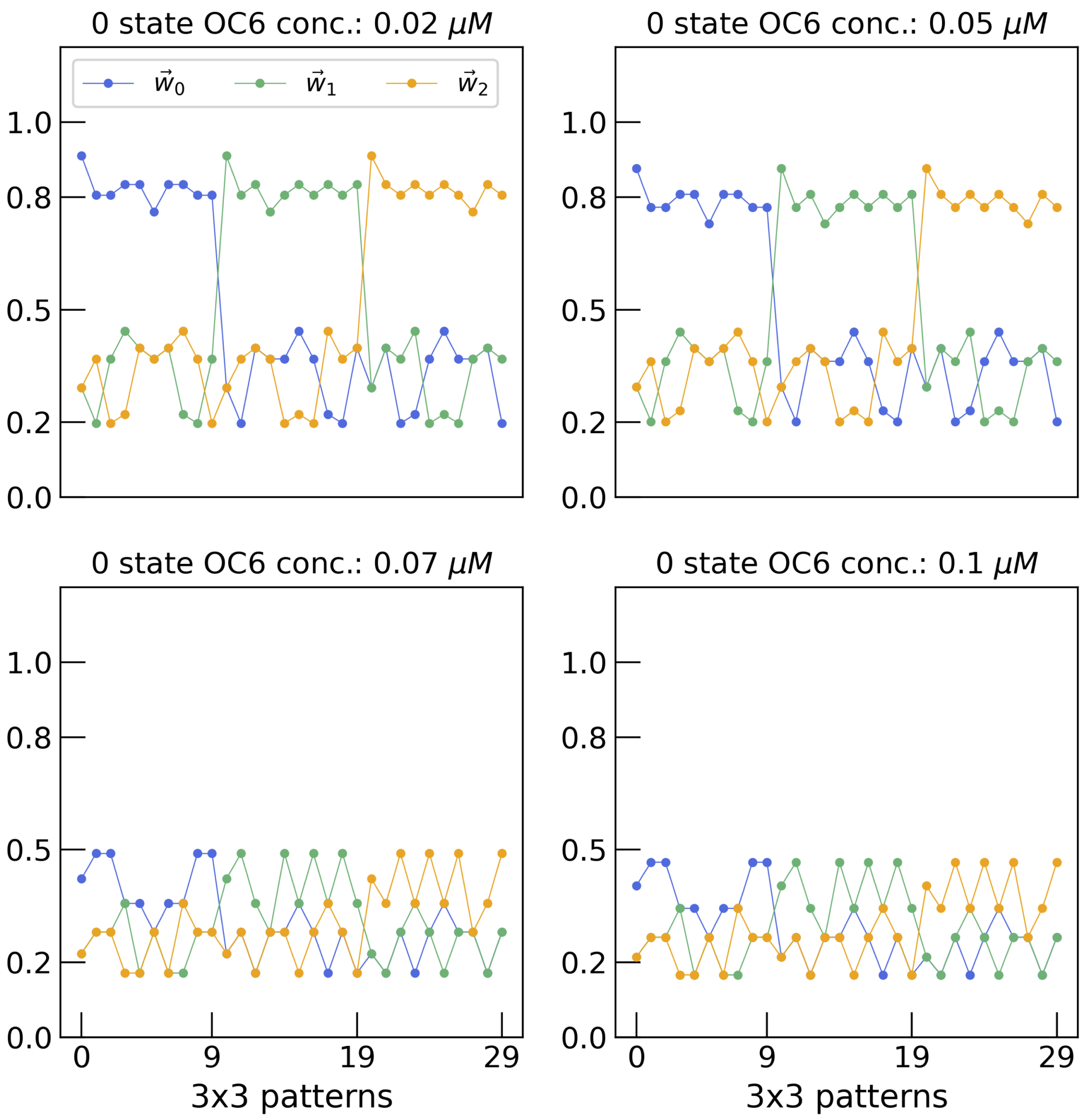

Supplementary Fig. 2: Simulated receiver output for 3×3-bit patterns. "0" state inducer concentration is varied from 0.02 μM to 0.1 μM.

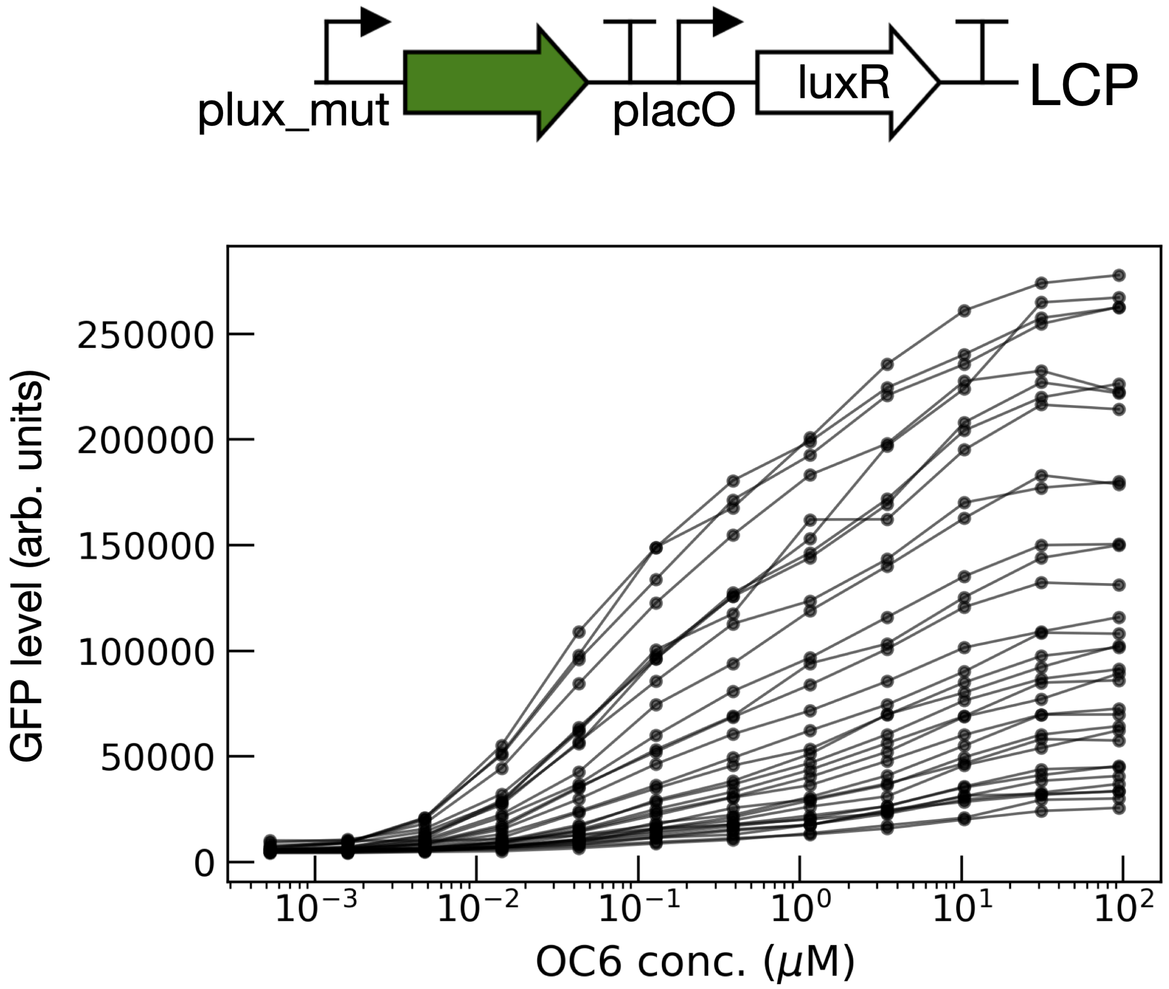

Supplementary Fig. 3: Promoter strength for genetic circuit with mutated P_{lux} promoters. The upper panel diagram shows the genetic circuit. Transcription factor LuxR is constitutively expressed driven by promoter P_{lacO}.

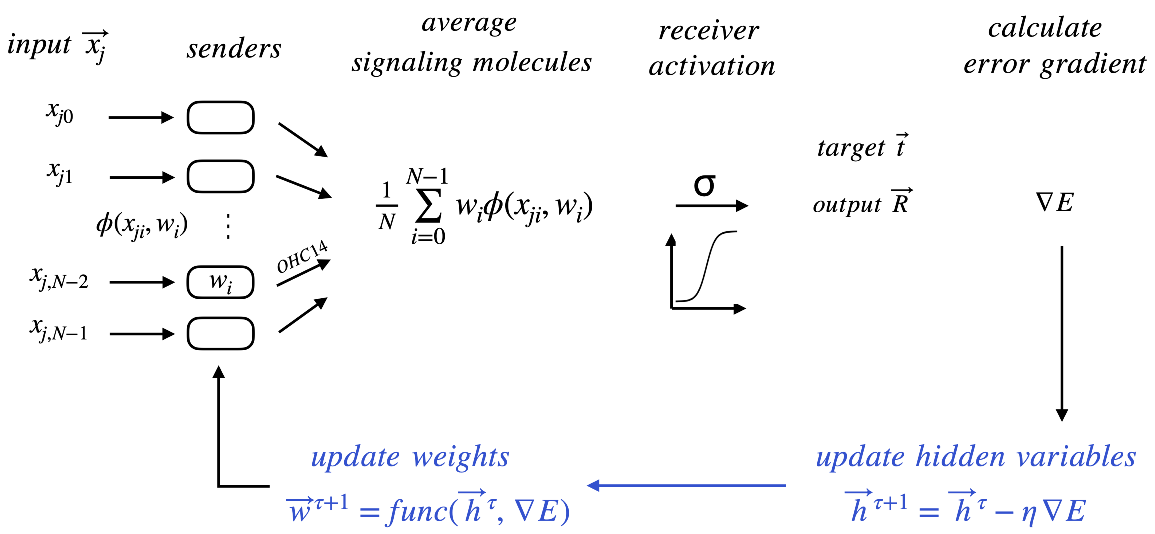

Supplementary Fig. 4: Adapted algorithm to update weights in discrete levels. Compared with Fig. 4a, an intermediate variable $\vec{h}$ is introduced for continuous update, while elements in $\vec{w}$ adopts new values only if corresponding h values reach one of the discrete weight levels.

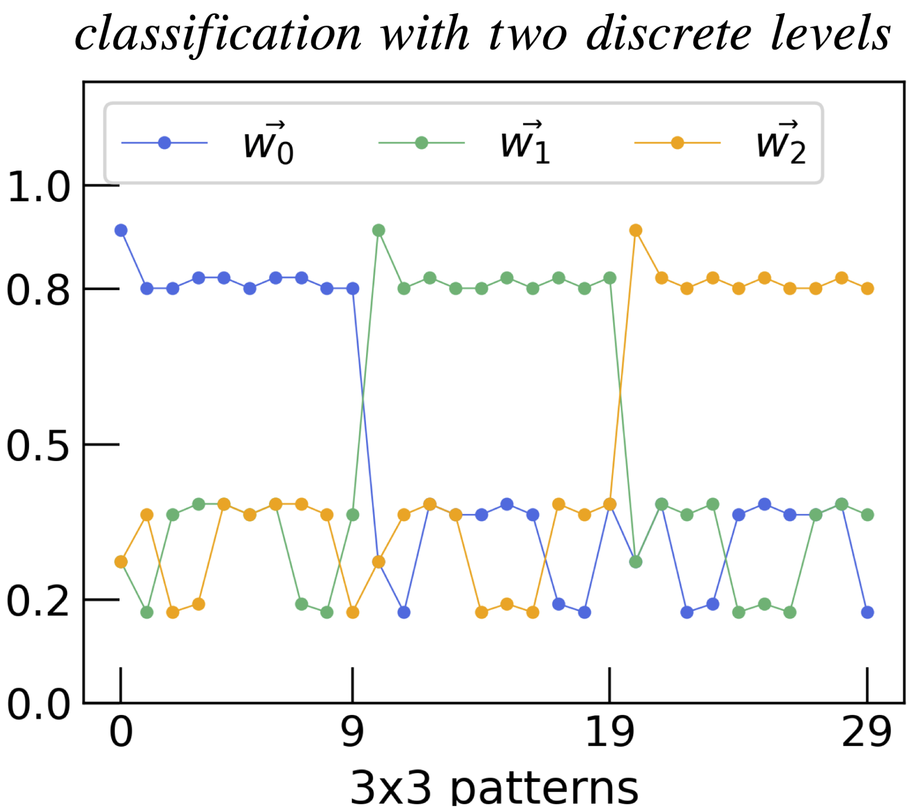

$$\vec{w}_0 = [1906, 1906, -735, -735, 1906, -735, -735, 1906, 1906]$$
$$\vec{w}_1 = [1906, -735, 1906, 1906, -735, 1906, -735, 1906, -735]$$
$$\vec{w}_2 = [-735, 1906, -735, 1906, -735, 1906, 1906, -735, 1906]$$

Supplementary Fig. 5: Classification of 3×3-bit patterns can be achieved using weights with only two distinct values.

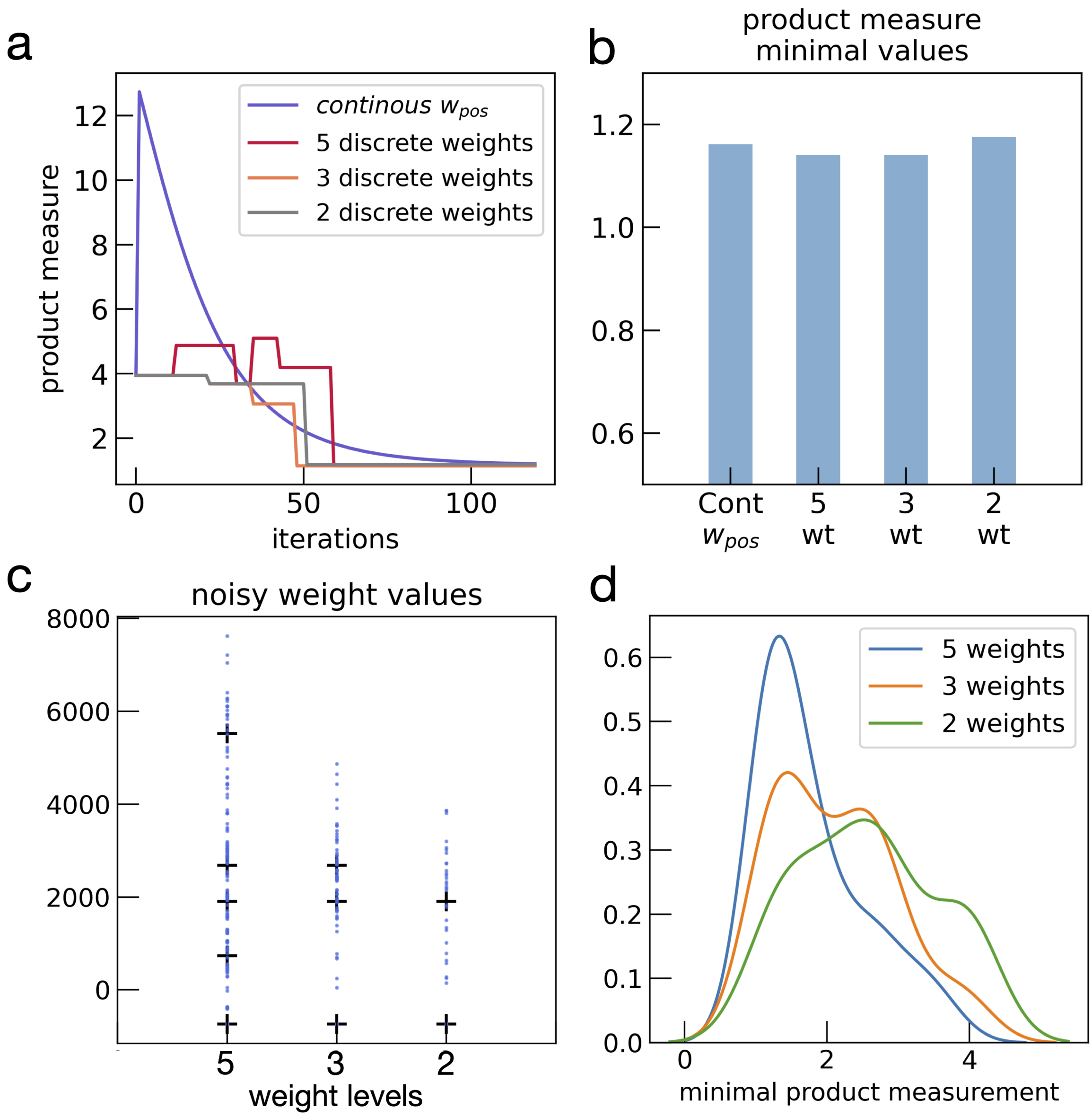

Supplementary Fig. 6: **a** Product measurement metric as in eq10 is updated across iterations for models using continuous positive weights, discrete weights with 5 distinct levels, 3 levels and 2 levels respectively. In all cases, weights are arbitarily initialized to negative values. The curve for continuous positive weights drops smoothly and curves for discrete weights show jagged edges. **b** Minimal product measurement is adopted as a metric for classification performance. A smaller value means better performance. Here we show the metric for models using continuous positive weights and discrete weights. **c** Noisy discrete weights for weight set contains 5, 3, and 2 distinct levels. Random weight values were generated around pre-selected weights (marked in '+') following a Gaussian distribution. For each randomized weight set, the minimal value of product measurement (eq10) was examined. The distribution of minimal product measurement is presented in **d** for varying weight levels. **d** As weight levels increase, the minimal product measurement exhibts a decreasing mean value with reduced variance, indicating improved classification performance.

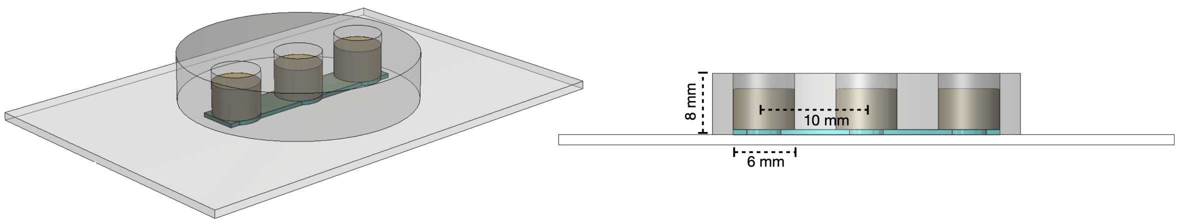

Supplementary Fig. 7: Layout of a fluidic device. The device is made of PDMS sealed on a glass slide, consisting of three wells on top of a channel. Each well is 6 mm in diameter and is 10 mm away from neighbor well(s). The channel is 100 μm in height (blue color). Each well can hold more than 150 μl solution. The 3D figures were generated using SolidWorks.

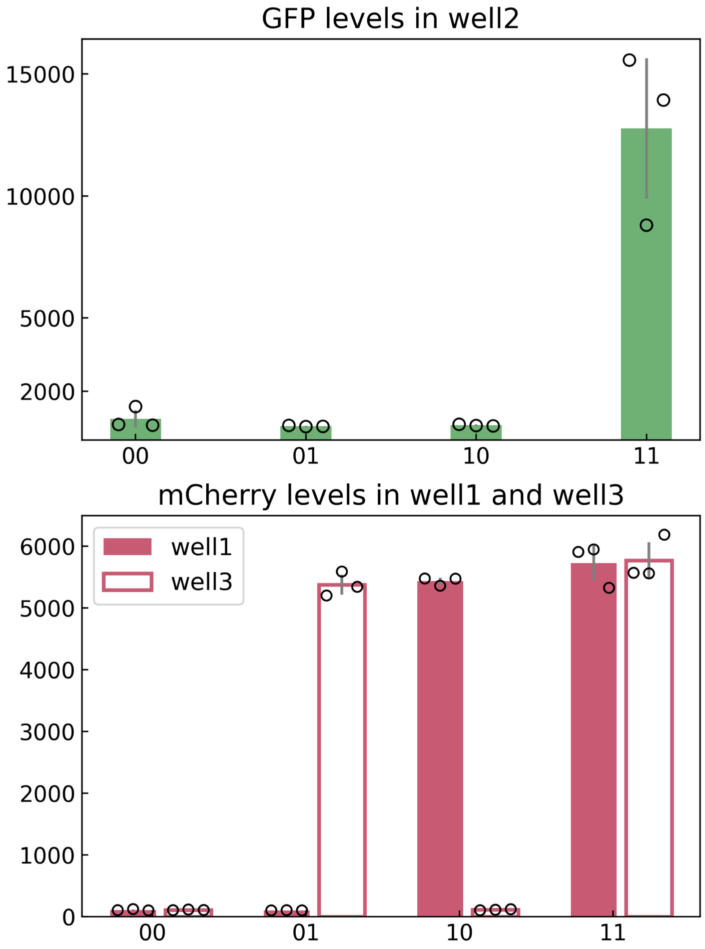

Supplementary Fig. 8: The three-well fluidic devices can classify 2-bit patterns. Experiments performed using equal weights (wild-type P_{lux}), effectively acting as an "AND" classifier. GFP levels in well2 for the four 2-bit patterns are presented as mean values of median EYFP ± SEM from separate devices (n=3). mCherry levels in well1 and well3 are presented as mean values of median levels ± SEM from three devices. Devices were autoclaved to eliminate contaminants after each experiment. More experimental detail is available in Supplementary Notes section Fluidics experiments.

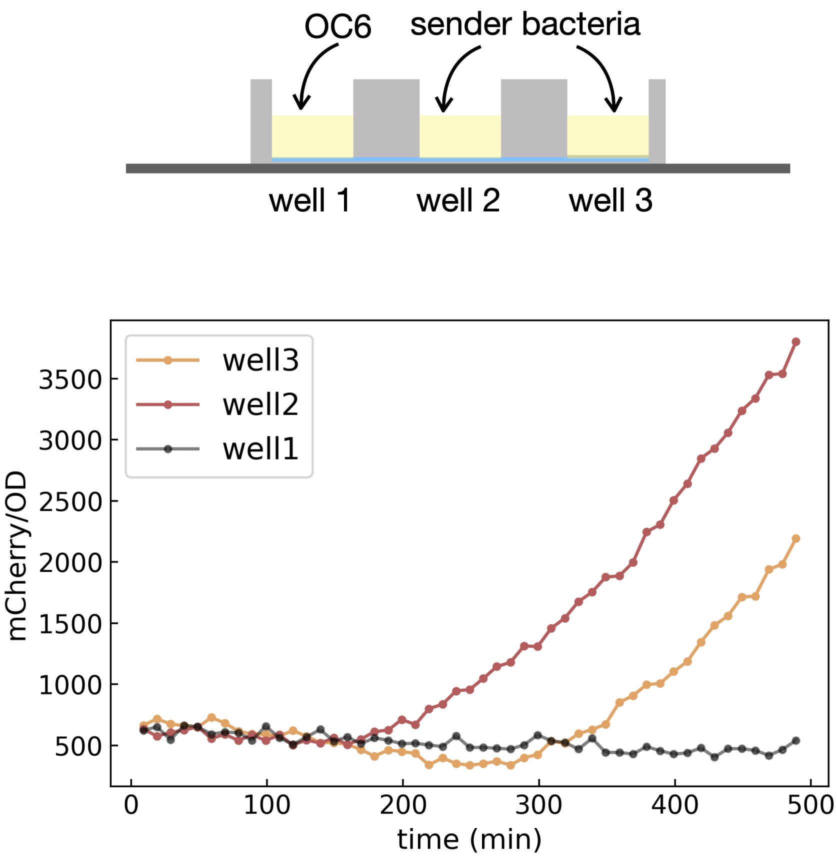

Supplementary Fig. 9: Experiment setup for the assay measured in plate-reader. The device was fixed on a plate-reader holder for measurement. OC6 (final concentration 10 μM) was added to well1. Senders (1 μl) from overnight bacteria solution were added to well2 and well3. Time courses of mCherry levels in three wells normalized to OD. Since well1 contains no bacteria, the mCherry level in well1 stays low and is presented as a control reference.

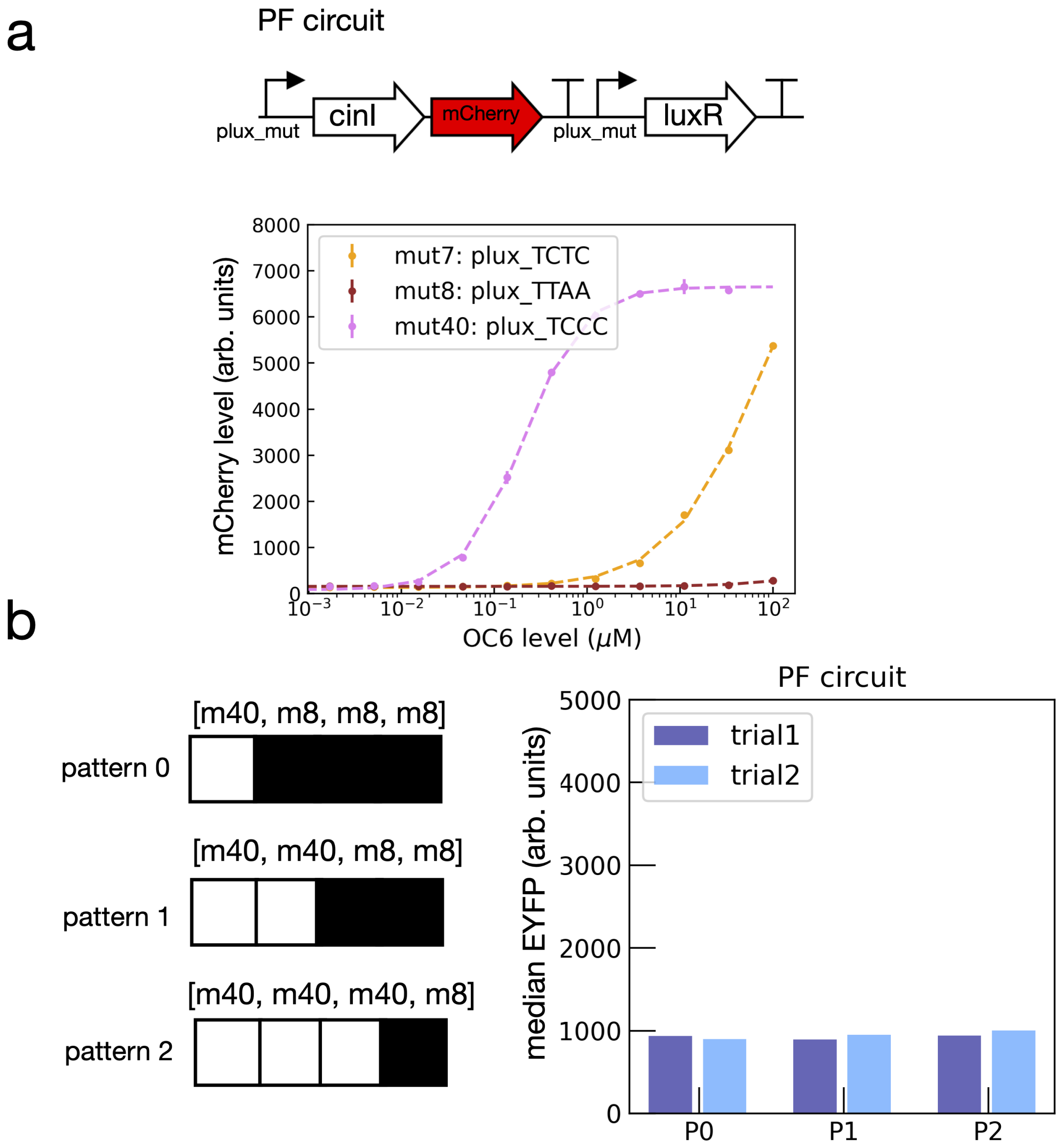

Supplementary Fig. 10: **Sender circuits with positive feedback regulation. a.** Genetic circuit diagram with positive feedback (PF) design and transfer functions of the PF circuits for three mutated P_{lux} promoters. Data are presented as mean values of median mCherry ± SEM from independent replicates (n=3). **b.** Patterns used in an assay to demonstrate that no cross-talks between senders have been observed. "m8" stands for P_{lux} mut8, a mutated P_{lux} promoter with weak strength. "m40" stands for P_{lux} mut40, a mutated P_{lux} promoter with high strength. All three patterns correspond to the sender mix that are supposed to result in low activation levels in receivers. In all three patterns, high concentrations of inducer OC6 were applied to P_{lux} mut8 only. If cross-talks occur, P_{lux} mut40 could become affected and produce OHC14 to activate receivers. As shown from the right figure, the median levels of receivers corresponding to the three patterns are consistently below 1000 a.u., indicating low activation levels in receivers.

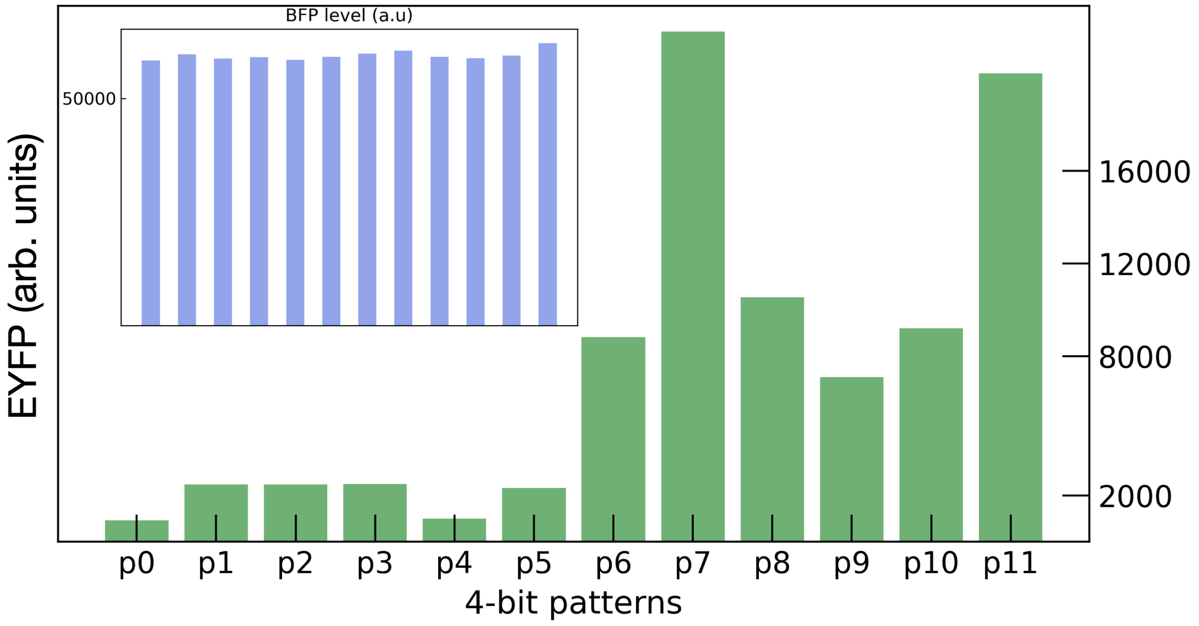

Supplementary Fig. 11: Senders with PF regulation were used for 4-bit patterns classification as in Fig. 3c. There is a clear distinction between receiver activation levels (presented in median EYFP) for patterns belong to two classes (p0-p5 vs. p6-p11). BFP levels in receivers are uniform across patterns, indicating the observed distinction in EYFP was not due to errors in mixing cell solutions.

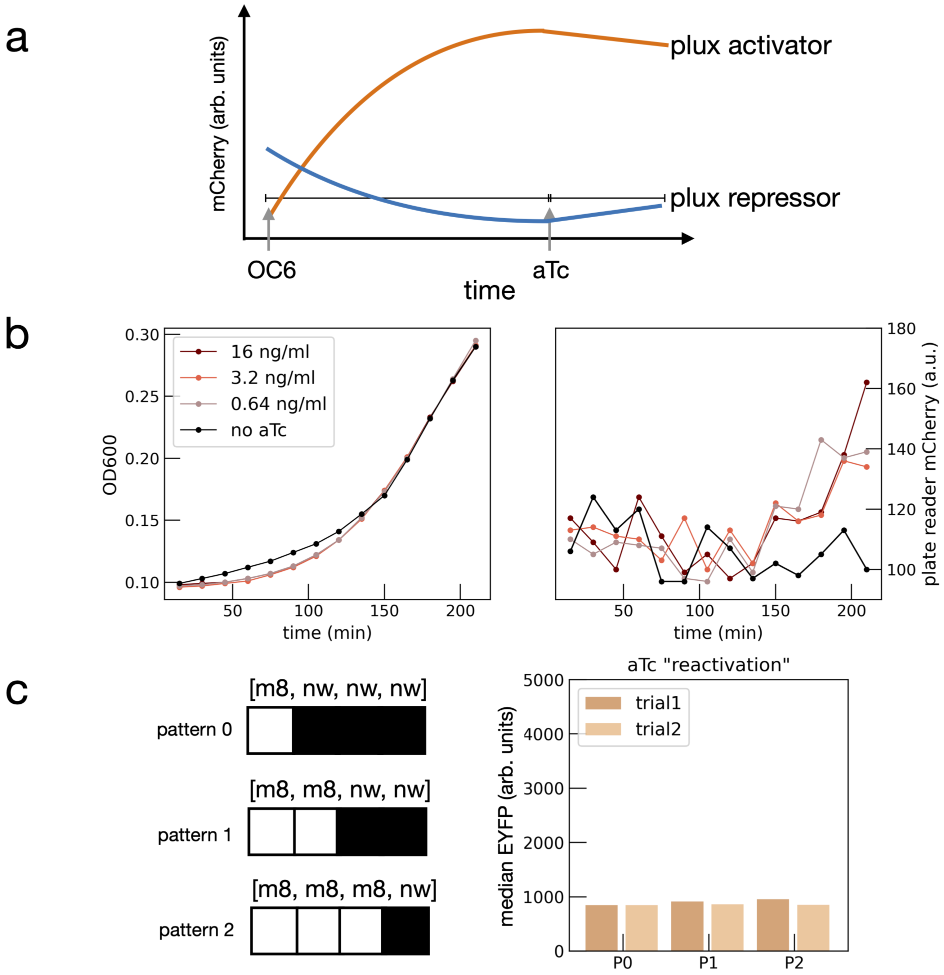

Supplementary Fig. 12: **Hypothetical effects of aTc on sender cell activities. a** Illustration of sender promoter activity across time for P_{lux} activator (orange curve) and P_{lux} repressor (blue curve) promoters. Upon adding OC6 ("1" bits), the activity of P_{lux} activator increases, producing increasing amount of mCherry. When aTc is introduced, P_{lux} activator promoter gradually decreases its activity due to P_{lacO} being turned off. The opposite trend is expected for the P_{lux} repressor promoter. **b** Time courses of cell growth (left) and bulk mCherry level (right) obtained from a plate reader for senders with the P_{lux} repressor promoter. These senders were incubated in OC6 (100 μM) for four hours and then diluted 100 times with varying concentrations of aTc (16, 3.2, 0.64 and 0 $\mathrm{ng\,ml^{-1}}$). The cell growth is not affected by aTc (left). However in the presence of aTc, senders start to show higher mCherry levels than without aTc at 150 min, indicating reactivation of P_{lux} repressor promoters. Considering a 15 min for mCherry maturation (FPbase [14]), we can conclude an incubation period in exposure to aTc upto approximately 120 min is safe enough to avoid the reactivation. **c** An assay to demonstrate that the protocol adopted in experiments does not lead to reactivation of senders with P_{lux} repressor promoter. Similar to Supplementary Fig. 10b, the three patterns are supposed to result in low receiver activation. In particular, "m8" stands for P_{lux} mut8, a P_{lux} mutation of weak promoter strength. "nw" stands for negative weight, i.e., P_{lux} repressor promoter. P_{lux} mut8 senders were incubated with aTc (20 $\mathrm{ng\,ml^{-1}}$), and P_{lux} repressor senders were incubated with OC6 (33 μM), following the protocol in Fig. 3b. As expected, the median EYFP levels in receivers corresponding to the three patterns are consistently below 1000 a.u., indicating low activation levels in receivers.

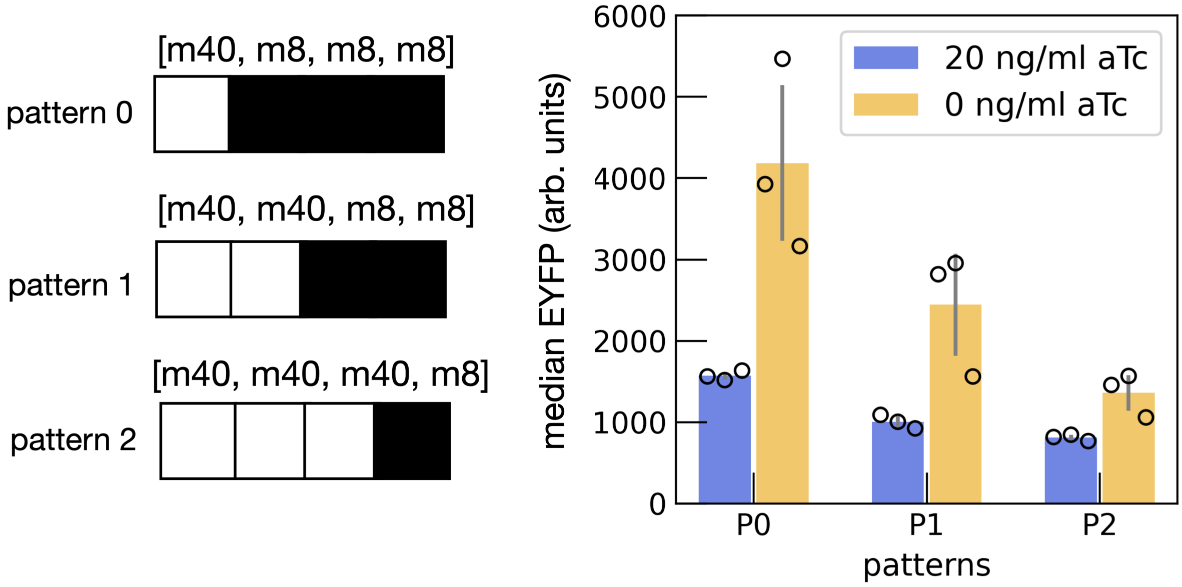

Supplementary Fig. 13: Demonstration of cross-talks between senders, using a similar procedure as in Supplementary Fig. 10b. Here sender circuits are in open loop as shown in Fig. 2a. Three patterns correspond to sender mix that is supposed to result in low activation levels in receivers. "m8" stands for P_{lux} mut8, a mutated P_{lux} promoter with weak strength. "m40" stands for P_{lux} mut40, a mutated P_{lux} promoter with strong strength. Averaged median levels of EYFP $\pm$ SEM are measured from receivers (n=3) in response to the three patterns, with or without aTc exposure. Senders incubation in aTc results in lower receiver activation (blue bars) than senders without aTc incubation (orange bars).

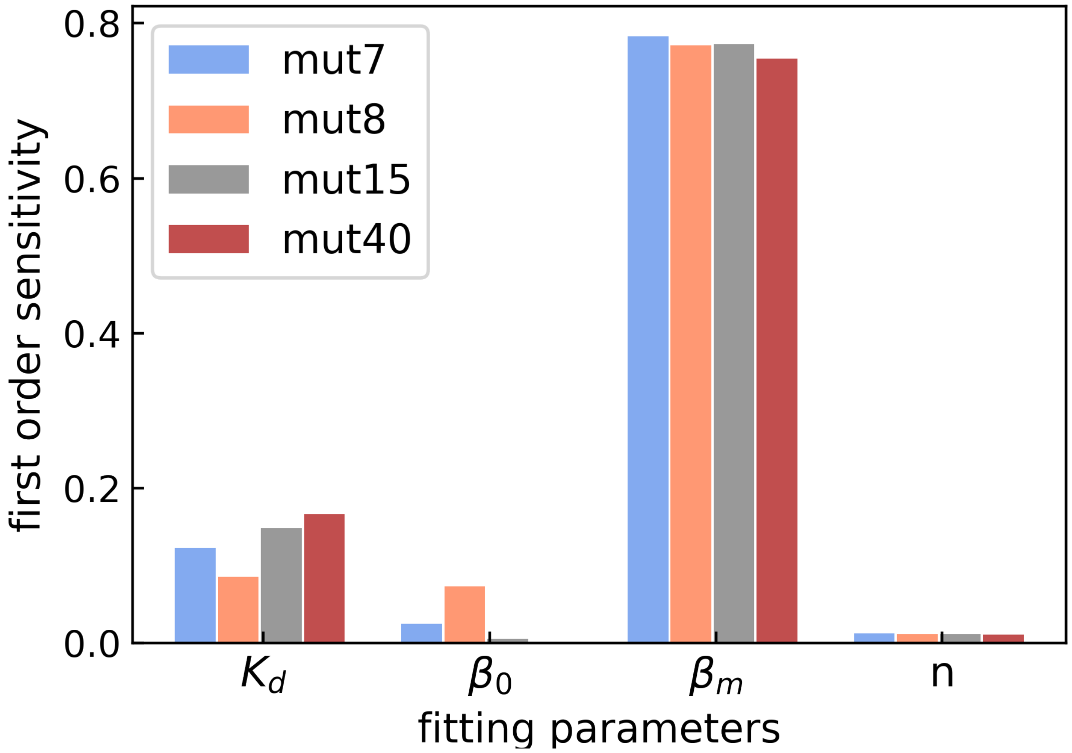

Supplementary Fig. 14: Sensitivity analysis of sender circuit transfer functions, which were fitted using Hill equations. Sensitivity analysis was carried out using a variance based method developed by Saltelli et al. [6]. The method decomposes variance of fitting errors by individual variables, giving rise to first order sensitivities. Sensitivity analysis was conducted for fitting of each mutated P_{lux} promoter. For four P_{lux} mutations, fitted transfer functions show highest sensitivities to the variable β_m, i.e., maximum promoter activity. Thus, we can simplify the corresponding transfer functions by setting parameters other than β_m as constant and single out β_m as the weight variable.

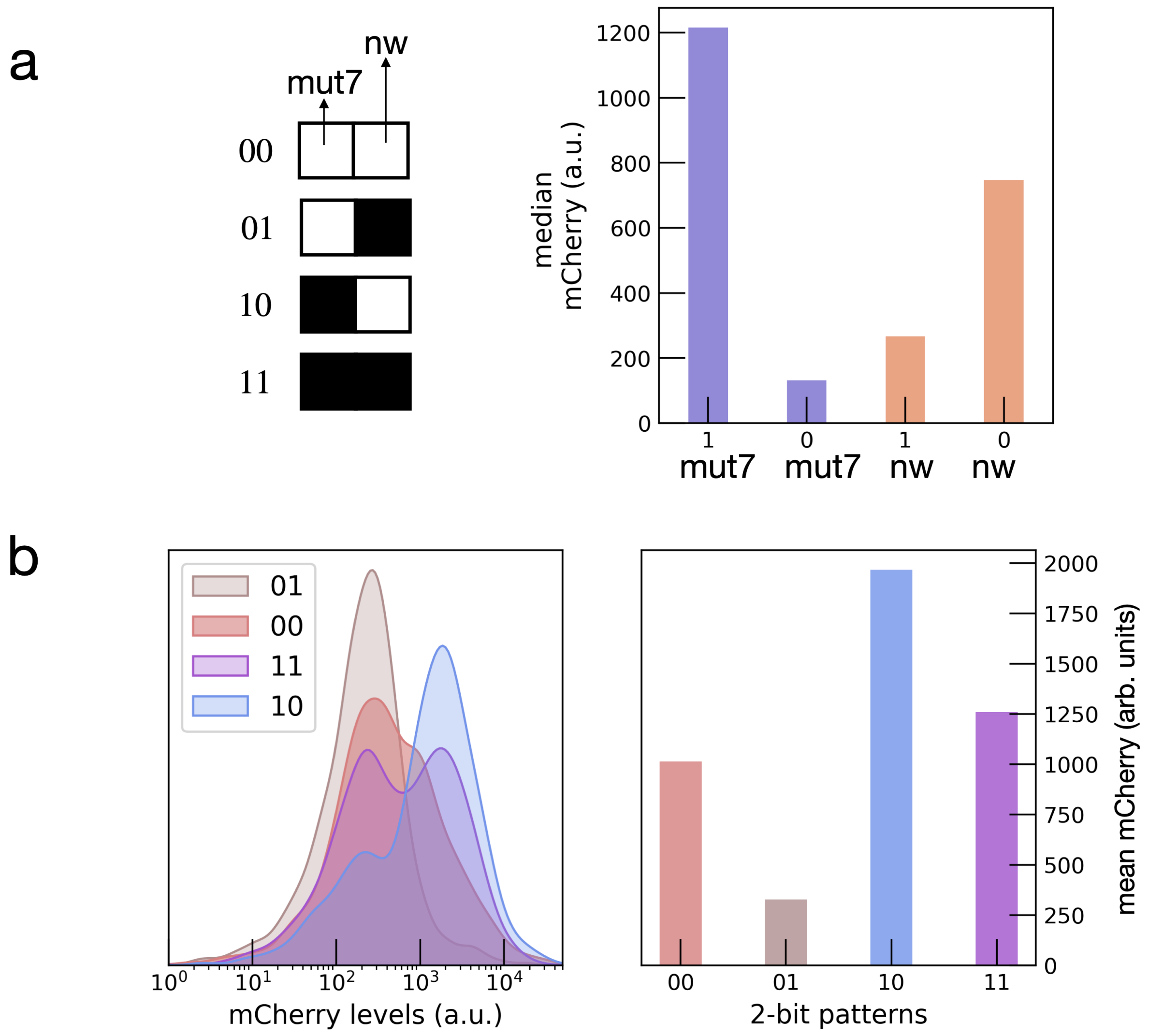

Supplementary Fig. 15: **Sender mix of P_{lux} repressor promoters (negative weight) and P_{lux} activator promoters (positive weight).** **a** (left) 2-bit patterns for mixing senders. The first bit represents the level of inducer OC6 for P_{lux} mut7 and the second bit for P_{lux} repressor promoter. (right) mCherry levels for individual sender to high ("1") and low ("0") inducer concentrations. "mut7" refers to senders with P_{lux} mut7 (purple color in the graph). "nw" refers to senders with P_{lux} repressor promoters with negative weight (orange color in the graph). mCherry levels were measured using FACS after senders being incubated for 210 min. **b** Histograms of measured mCherry when senders were mixed according to the 2-bit patterns. Average mCherry levels are shown on the right. From the data, we can tell that the effect of negative weight senders on the overall mCherry level depends on input patterns.

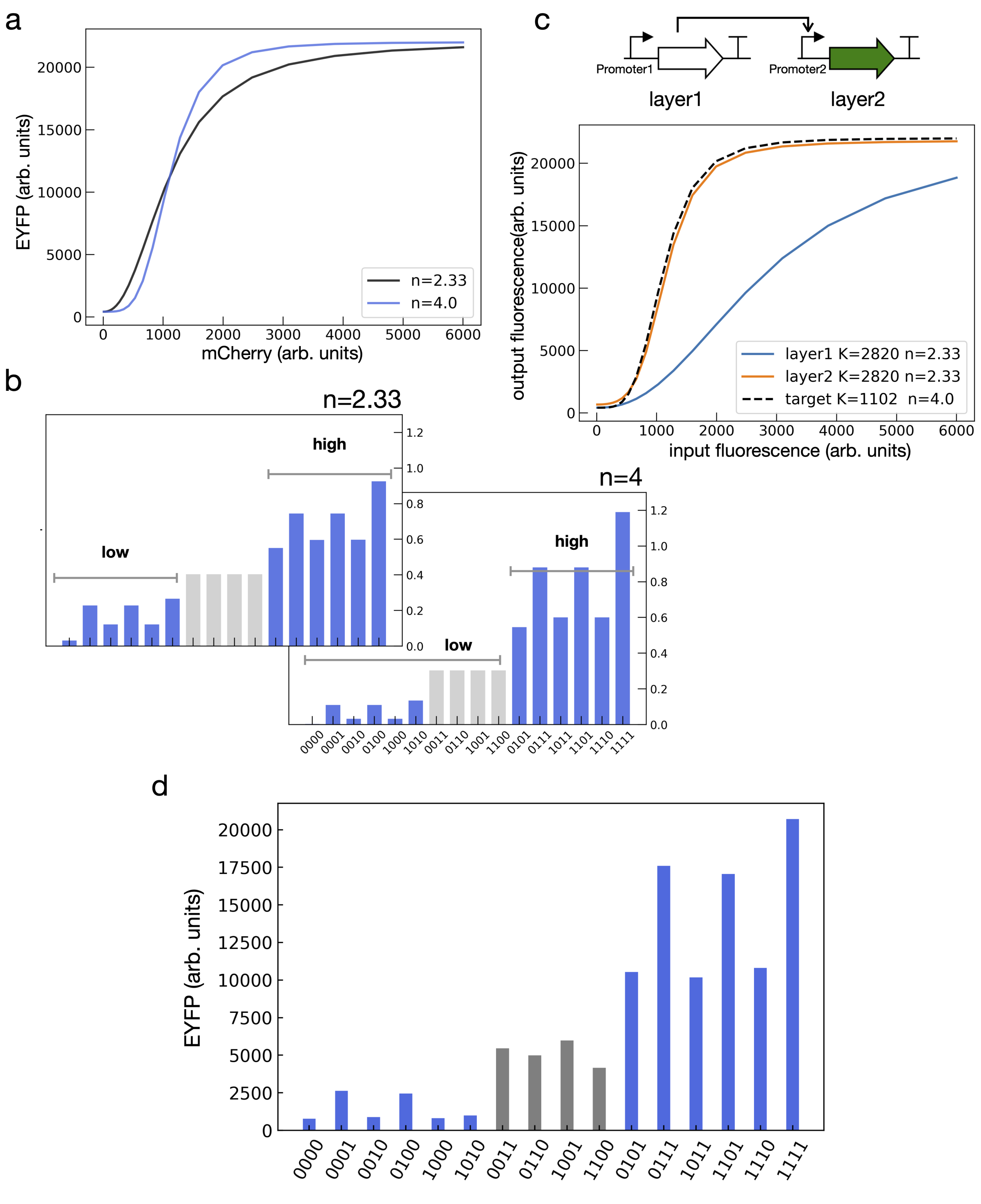

a
EYFP (arb. units)
mCherry (arb. units)
n=2.33
n=4.0
b
n=2.33
high
low
n=4
0000
0001
0010
0100
1000
1010
0011
0110
1001
1100
0101
0111
1011
1101
1110
1111
c
Promoter1
layer1
Promoter2
layer2
output fluorescence(arb. units)
input fluorescence (arb. units)
layer1 K=2820 n=2.33
layer2 K=2820 n=2.33
target K=1102 n=4.0
d
EYFP (arb. units)

Supplementary Fig. 16: **a** Classification outcome can be adjusted by tuning the receiver transfer function. Based on simulations, the receiver transfer function becomes sharpened as n increases from 2.33 to 4. Here the black curve is fitted using the Hill equation to experimental measurement as in Fig. 4c with n equals 2.33. **b.** Simulated classification of the 16 patterns is improved as n increases from 2.33 to 4. (Upper left) When n equals 2.33, the bars in grey color are in an intermediate category and cannot be readily classified into either "high" or "low" patterns. (Lower right) When n equals 4, the grey bars are more separate from the "high" category and can be classified as "low". **c.** Ultra-sensitivity in receiver transfer function can be achieved by cascading two layers of genetic circuits. The curves are simulated results. **d.** Experimentally measured receiver output presented in median EYFP levels for the complete 16 patterns. The blue bars correspond to the 12 patterns shown in Fig. 3c. The additional 4 patterns are in grey.

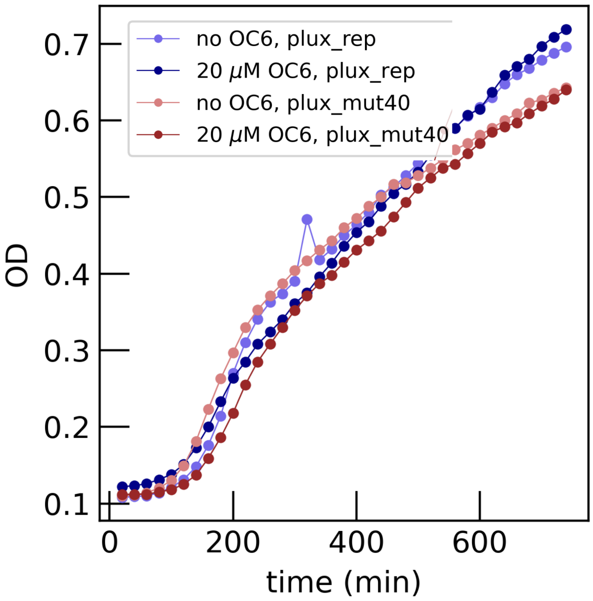

Supplementary Fig. 17: Time courses of cell growth measured in OD600. The measurements were obtained from a plate reader for senders with P_{lux} repressor and P_{lux} mut40 promoter, incubated either with or without OC6. Cells were diluted 100 times from overnight culture.

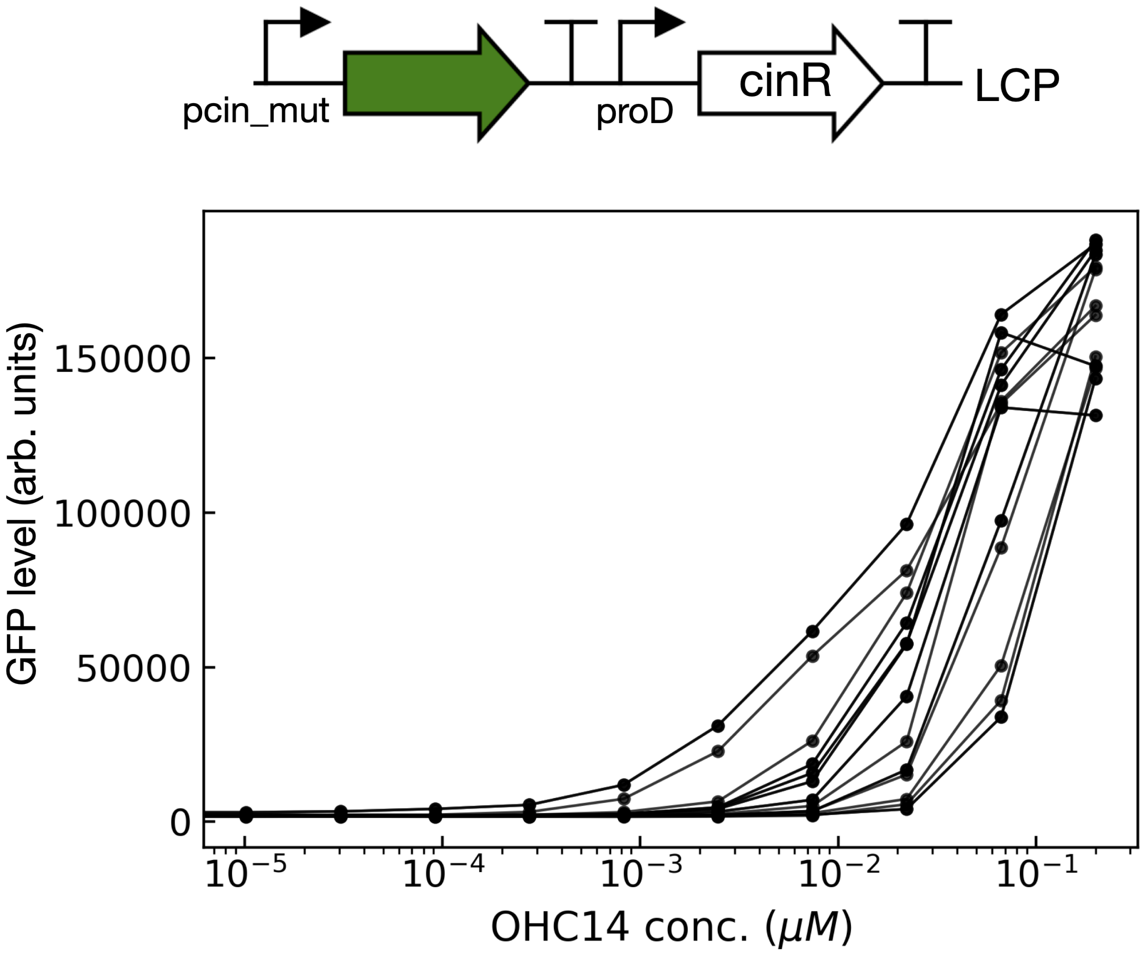

Supplementary Fig. 18: Promoter strength for genetic circuit with mutated P_{cin} promoters. The upper panel diagram shows the genetic circuit. Transcription factor CinR is constitutively expressed under the regulation of promoter P_{roD}. The first three base pairs in P_{cin} promoter operator region are mutated, leading to varying thresholds in transfer functions. The maximum levels of the transfer functions are not significantly affected, which is different from the outcome as in Supplementary Fig. 3 when mutating P_{lux} promoter.

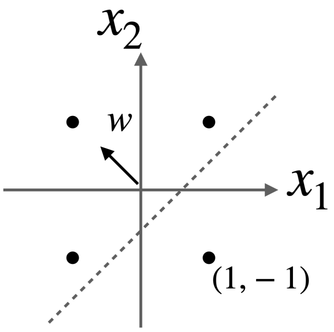

$\vec{w} = (-1,1)\ b = 0.5$

(x_1, x_2)	$(1,1)$	$(-1,1)$	$(-1,-1)$	$(1,-1)$
y	0.5	2.5	0.5	– 1.5

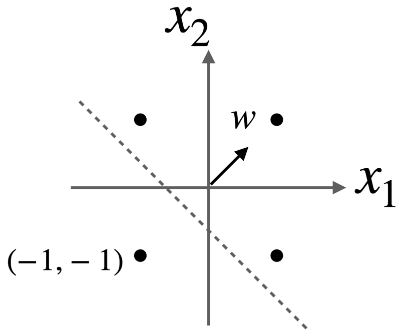

$\vec{w} = (1,1)\ b = 0.5$

(x_1, x_2)	$(1,1)$	$(-1,1)$	$(-1,-1)$	$(1,-1)$
y	2.5	0.5	– 1.5	0.5

Supplementary Fig. 19: An illustration of the notion that negative weight value is essential to classify certain patterns. Weight vector (-1,1) can separate the point (1,-1) from the other three points (1,1), (-1,1) and (-1,-1). Weight vector (1,1) contains no negative value and shows a decision boundary not in the desired direction to satisfy the classification target.

$\vec{w}_0 \circ \vec{p}_0$	0 1 0 4 4 0	
$\vec{w}_0 \circ \vec{p}_1$	0 1 1 3 4 0	
$\vec{w}_0 \circ \vec{p}_2$	0 1 1 3 4 0	$= \vec{w}_0 \circ \vec{p}_1$
$\vec{w}_0 \circ \vec{p}_3$	0 1 0 4 3 1	
$\vec{w}_0 \circ \vec{p}_4$	0 1 0 4 3 1	$= \vec{w}_0 \circ \vec{p}_3$
$\vec{w}_0 \circ \vec{p}_5$	1 0 0 4 4 0	
$\vec{w}_0 \circ \vec{p}_6$	0 1 0 4 3 1	$= \vec{w}_0 \circ \vec{p}_3$
$\vec{w}_0 \circ \vec{p}_7$	0 1 0 4 3 1	$= \vec{w}_0 \circ \vec{p}_3$
$\vec{w}_0 \circ \vec{p}_8$	0 1 1 3 4 0	$= \vec{w}_0 \circ \vec{p}_1$
$\vec{w}_0 \circ \vec{p}_9$	0 1 1 3 4 0	$= \vec{w}_0 \circ \vec{p}_1$
$\vec{w}_0 \circ \vec{p}_{10}$	1 0 2 2 1 3	
$\vec{w}_0 \circ \vec{p}_{11}$	1 0 3 1 1 3	
$\vec{w}_0 \circ \vec{p}_{12}$	1 0 1 3 1 3	
$\vec{w}_0 \circ \vec{p}_{13}$	1 0 2 2 2 2	
$\vec{w}_0 \circ \vec{p}_{14}$	1 0 2 2 2 2	$= \vec{w}_0 \circ \vec{p}_{13}$
$\vec{w}_0 \circ \vec{p}_{15}$	0 1 2 2 1 3	
$\vec{w}_0 \circ \vec{p}_{16}$	1 0 2 2 2 2	$= \vec{w}_0 \circ \vec{p}_{13}$
$\vec{w}_0 \circ \vec{p}_{17}$	1 0 2 2 0 4	
$\vec{w}_0 \circ \vec{p}_{18}$	1 0 3 1 1 3	$= \vec{w}_0 \circ \vec{p}_{11}$
$\vec{w}_0 \circ \vec{p}_{19}$	1 0 1 3 1 3	$= \vec{w}_0 \circ \vec{p}_{12}$
$\vec{w}_0 \circ \vec{p}_{20}$	1 0 2 2 1 3	$= \vec{w}_0 \circ \vec{p}_{10}$
$\vec{w}_0 \circ \vec{p}_{21}$	1 0 1 3 1 3	$= \vec{w}_0 \circ \vec{p}_{12}$
$\vec{w}_0 \circ \vec{p}_{22}$	1 0 3 1 1 3	$= \vec{w}_0 \circ \vec{p}_{11}$
$\vec{w}_0 \circ \vec{p}_{23}$	1 0 2 2 0 4	$= \vec{w}_0 \circ \vec{p}_{17}$
$\vec{w}_0 \circ \vec{p}_{24}$	1 0 2 2 2 2	$= \vec{w}_0 \circ \vec{p}_{13}$
$\vec{w}_0 \circ \vec{p}_{25}$	0 1 2 2 1 3	$= \vec{w}_0 \circ \vec{p}_{15}$
$\vec{w}_0 \circ \vec{p}_{26}$	1 0 2 2 2 2	$= \vec{w}_0 \circ \vec{p}_{13}$
$\vec{w}_0 \circ \vec{p}_{27}$	1 0 2 2 2 2	$= \vec{w}_0 \circ \vec{p}_{13}$
$\vec{w}_0 \circ \vec{p}_{28}$	1 0 1 3 1 3	$= \vec{w}_0 \circ \vec{p}_{12}$
$\vec{w}_0 \circ \vec{p}_{29}$	1 0 3 1 1 3	$= \vec{w}_0 \circ \vec{p}_{11}$

$\vec{w}_1 \circ \vec{p}_0$	1 0 2 2 1 3	$= \vec{w}_0 \circ \vec{p}_{10}$
$\vec{w}_1 \circ \vec{p}_1$	1 0 3 1 1 3	$= \vec{w}_0 \circ \vec{p}_{11}$
$\vec{w}_1 \circ \vec{p}_2$	1 0 2 2 2 2	$= \vec{w}_0 \circ \vec{p}_{13}$
$\vec{w}_1 \circ \vec{p}_3$	0 1 2 2 1 3	$= \vec{w}_0 \circ \vec{p}_{15}$
$\vec{w}_1 \circ \vec{p}_4$	1 0 1 3 1 3	$= \vec{w}_0 \circ \vec{p}_{12}$
$\vec{w}_1 \circ \vec{p}_5$	1 0 2 2 2 2	$= \vec{w}_0 \circ \vec{p}_{13}$
$\vec{w}_1 \circ \vec{p}_6$	1 0 1 3 1 3	$= \vec{w}_0 \circ \vec{p}_{12}$
$\vec{w}_1 \circ \vec{p}_7$	1 0 2 2 0 4	$= \vec{w}_0 \circ \vec{p}_{17}$
$\vec{w}_1 \circ \vec{p}_8$	1 0 3 1 1 3	$= \vec{w}_0 \circ \vec{p}_{11}$
$\vec{w}_1 \circ \vec{p}_9$	1 0 2 2 2 2	$= \vec{w}_0 \circ \vec{p}_{13}$
$\vec{w}_1 \circ \vec{p}_{10}$	0 1 0 4 4 0	$= \vec{w}_0 \circ \vec{p}_0$
$\vec{w}_1 \circ \vec{p}_{11}$	0 1 1 3 4 0	$= \vec{w}_0 \circ \vec{p}_1$
$\vec{w}_1 \circ \vec{p}_{12}$	0 1 0 4 3 1	$= \vec{w}_0 \circ \vec{p}_3$
$\vec{w}_1 \circ \vec{p}_{13}$	1 0 0 4 4 0	$= \vec{w}_0 \circ \vec{p}_5$
$\vec{w}_1 \circ \vec{p}_{14}$	0 1 1 3 4 0	$= \vec{w}_0 \circ \vec{p}_1$
$\vec{w}_1 \circ \vec{p}_{15}$	0 1 0 4 3 1	$= \vec{w}_0 \circ \vec{p}_3$
$\vec{w}_1 \circ \vec{p}_{16}$	0 1 1 3 4 0	$= \vec{w}_0 \circ \vec{p}_1$
$\vec{w}_1 \circ \vec{p}_{17}$	0 1 0 4 3 1	$= \vec{w}_0 \circ \vec{p}_3$
$\vec{w}_1 \circ \vec{p}_{18}$	0 1 1 3 4 0	$= \vec{w}_0 \circ \vec{p}_1$
$\vec{w}_1 \circ \vec{p}_{19}$	0 1 0 4 3 1	$= \vec{w}_0 \circ \vec{p}_3$
$\vec{w}_1 \circ \vec{p}_{20}$	1 0 2 2 1 3	$= \vec{w}_0 \circ \vec{p}_{10}$
$\vec{w}_1 \circ \vec{p}_{21}$	1 0 1 3 1 3	$= \vec{w}_0 \circ \vec{p}_{12}$
$\vec{w}_1 \circ \vec{p}_{22}$	1 0 2 2 2 2	$= \vec{w}_0 \circ \vec{p}_{13}$
$\vec{w}_1 \circ \vec{p}_{23}$	0 1 2 2 1 3	$= \vec{w}_0 \circ \vec{p}_{15}$
$\vec{w}_1 \circ \vec{p}_{24}$	1 0 3 1 1 3	$= \vec{w}_0 \circ \vec{p}_{11}$
$\vec{w}_1 \circ \vec{p}_{25}$	1 0 2 2 0 4	$= \vec{w}_0 \circ \vec{p}_{17}$
$\vec{w}_1 \circ \vec{p}_{26}$	1 0 3 1 1 3	$= \vec{w}_0 \circ \vec{p}_{11}$
$\vec{w}_1 \circ \vec{p}_{27}$	1 0 2 2 2 2	$= \vec{w}_0 \circ \vec{p}_{13}$
$\vec{w}_1 \circ \vec{p}_{28}$	1 0 1 3 1 3	$= \vec{w}_0 \circ \vec{p}_{12}$
$\vec{w}_1 \circ \vec{p}_{29}$	1 0 2 2 2 2	$= \vec{w}_0 \circ \vec{p}_{13}$

$\vec{w}_2 \circ \vec{p}_0$	1 0 2 2 1 3	$= \vec{w}_0 \circ \vec{p}_{10}$
$\vec{w}_2 \circ \vec{p}_1$	1 0 2 2 2 2	$= \vec{w}_0 \circ \vec{p}_{13}$
$\vec{w}_2 \circ \vec{p}_2$	1 0 3 1 1 3	$= \vec{w}_0 \circ \vec{p}_{11}$
$\vec{w}_2 \circ \vec{p}_3$	1 0 2 2 0 4	$= \vec{w}_0 \circ \vec{p}_{17}$
$\vec{w}_2 \circ \vec{p}_4$	1 0 1 3 1 3	$= \vec{w}_0 \circ \vec{p}_{12}$
$\vec{w}_2 \circ \vec{p}_5$	1 0 2 2 2 2	$= \vec{w}_0 \circ \vec{p}_{13}$
$\vec{w}_2 \circ \vec{p}_6$	1 0 1 3 1 3	$= \vec{w}_0 \circ \vec{p}_{12}$
$\vec{w}_2 \circ \vec{p}_7$	0 1 2 2 1 3	$= \vec{w}_0 \circ \vec{p}_{15}$
$\vec{w}_2 \circ \vec{p}_8$	1 0 2 2 2 2	$= \vec{w}_0 \circ \vec{p}_{13}$
$\vec{w}_2 \circ \vec{p}_9$	1 0 3 1 1 3	$= \vec{w}_0 \circ \vec{p}_{11}$
$\vec{w}_2 \circ \vec{p}_{10}$	1 0 2 2 1 3	$= \vec{w}_0 \circ \vec{p}_{10}$
$\vec{w}_2 \circ \vec{p}_{11}$	1 0 2 2 2 2	$= \vec{w}_0 \circ \vec{p}_{13}$
$\vec{w}_2 \circ \vec{p}_{12}$	1 0 1 3 1 3	$= \vec{w}_0 \circ \vec{p}_{12}$
$\vec{w}_2 \circ \vec{p}_{13}$	1 0 2 2 2 2	$= \vec{w}_0 \circ \vec{p}_{13}$
$\vec{w}_2 \circ \vec{p}_{14}$	1 0 3 1 1 3	$= \vec{w}_0 \circ \vec{p}_{11}$
$\vec{w}_2 \circ \vec{p}_{15}$	1 0 2 2 0 4	$= \vec{w}_0 \circ \vec{p}_{17}$
$\vec{w}_2 \circ \vec{p}_{16}$	1 0 3 1 1 3	$= \vec{w}_0 \circ \vec{p}_{11}$
$\vec{w}_2 \circ \vec{p}_{17}$	0 1 2 2 1 3	$= \vec{w}_0 \circ \vec{p}_{15}$
$\vec{w}_2 \circ \vec{p}_{18}$	1 0 2 2 2 2	$= \vec{w}_0 \circ \vec{p}_{13}$
$\vec{w}_2 \circ \vec{p}_{19}$	1 0 1 3 1 3	$= \vec{w}_0 \circ \vec{p}_{12}$
$\vec{w}_2 \circ \vec{p}_{20}$	0 1 0 4 4 0	$= \vec{w}_0 \circ \vec{p}_0$
$\vec{w}_2 \circ \vec{p}_{21}$	0 1 0 4 3 1	$= \vec{w}_0 \circ \vec{p}_3$
$\vec{w}_2 \circ \vec{p}_{22}$	0 1 1 3 4 0	$= \vec{w}_0 \circ \vec{p}_1$
$\vec{w}_2 \circ \vec{p}_{23}$	0 1 0 4 3 1	$= \vec{w}_0 \circ \vec{p}_3$
$\vec{w}_2 \circ \vec{p}_{24}$	0 1 1 3 4 0	$= \vec{w}_0 \circ \vec{p}_1$
$\vec{w}_2 \circ \vec{p}_{25}$	0 1 0 4 3 1	$= \vec{w}_0 \circ \vec{p}_3$
$\vec{w}_2 \circ \vec{p}_{26}$	0 1 1 3 4 0	$= \vec{w}_0 \circ \vec{p}_1$
$\vec{w}_2 \circ \vec{p}_{27}$	1 0 0 4 4 0	$= \vec{w}_0 \circ \vec{p}_5$
$\vec{w}_2 \circ \vec{p}_{28}$	0 1 0 4 3 1	$= \vec{w}_0 \circ \vec{p}_3$
$\vec{w}_2 \circ \vec{p}_{29}$	0 1 1 3 4 0	$= \vec{w}_0 \circ \vec{p}_1$

Supplementary Fig. 20: Lists of products of weights and 3×3-bit patterns. Left matrix contains the element-wise product of $\vec{w}_0$ and each pattern. Middle and right matrices are the same as the left matrix but for $\vec{w}_1$ and $\vec{w}_2$. In each matrix, the numerical numbers in each row are coefficients of the product $\vec{w} \circ \vec{p}$ with respect to mutated P_{lux} promoters. The coefficients are in the order of m15, m15*, m7, m7*, nw, nw*, where * represents incubation with OC6. For example, as in Table S3, $\vec{w}_0$=[m7, m7, nw, nw, m15, nw, nw, m7, m7] and $\vec{p}_0$ is= [1, 1, 0, 0, 1, 0, 0, 1, 1]. The element-wise product is [m7*, m7*, nw, nw, m15*, nw, nw, m7*, m7*], the sum of which equals 0m15+1m15*+0m7+4m7*+4nw+0nw*. The coefficients [0, 1, 0, 4, 4, 0] are thus with the entries in the first row of the left matrix. Notice that only ten rows in the left matrix are unique, corresponding to the ten distinct levels in Fig. 5b lower panel graphs. All other element-wise products are repeats of these ten products.

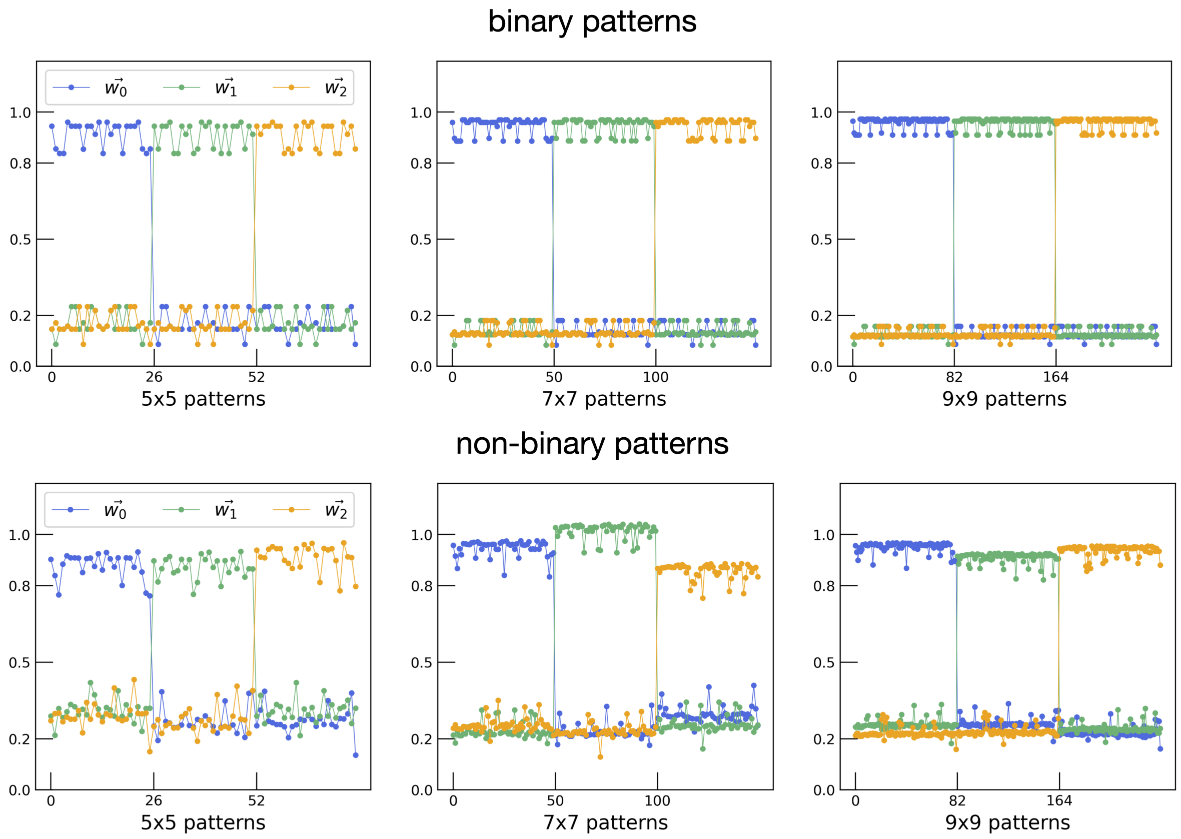

Supplementary Fig. 21: Simulated results for classifying 5×5, 7×7 and 9×9-bit patterns using continuous positive weight levels. The same patterns as in Fig. 6 were used for classification. (upper) Simulated results for binary patterns. (lower) Simulated results for non-binary patterns.

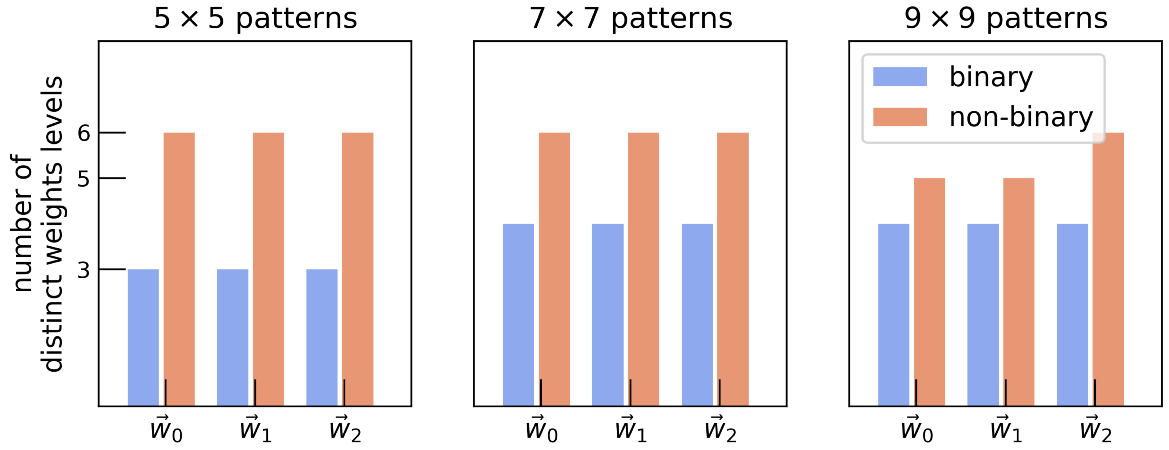

Supplementary Fig. 22: Number of distinct weight levels involved in classification as in Fig. 6 for 5×5, 7×7 and 9×9-bit patterns. Blue bars are numbers of discrete weight levels involved to classify binary patterns and red bars are for non-binary patterns.

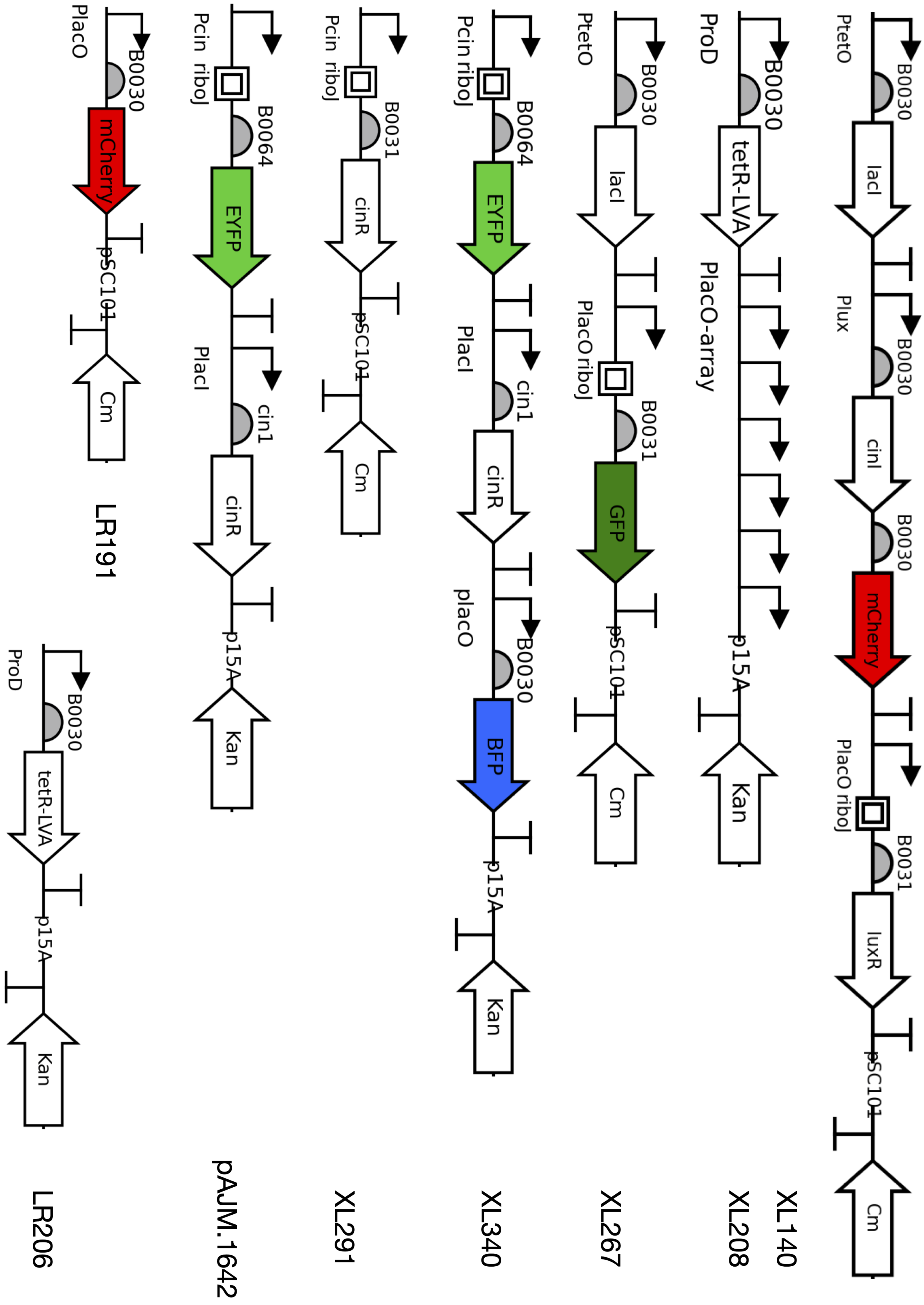

Supplementary Fig. 23: Plasmid maps used in experiments

2.1 Fluorescence histogram figures

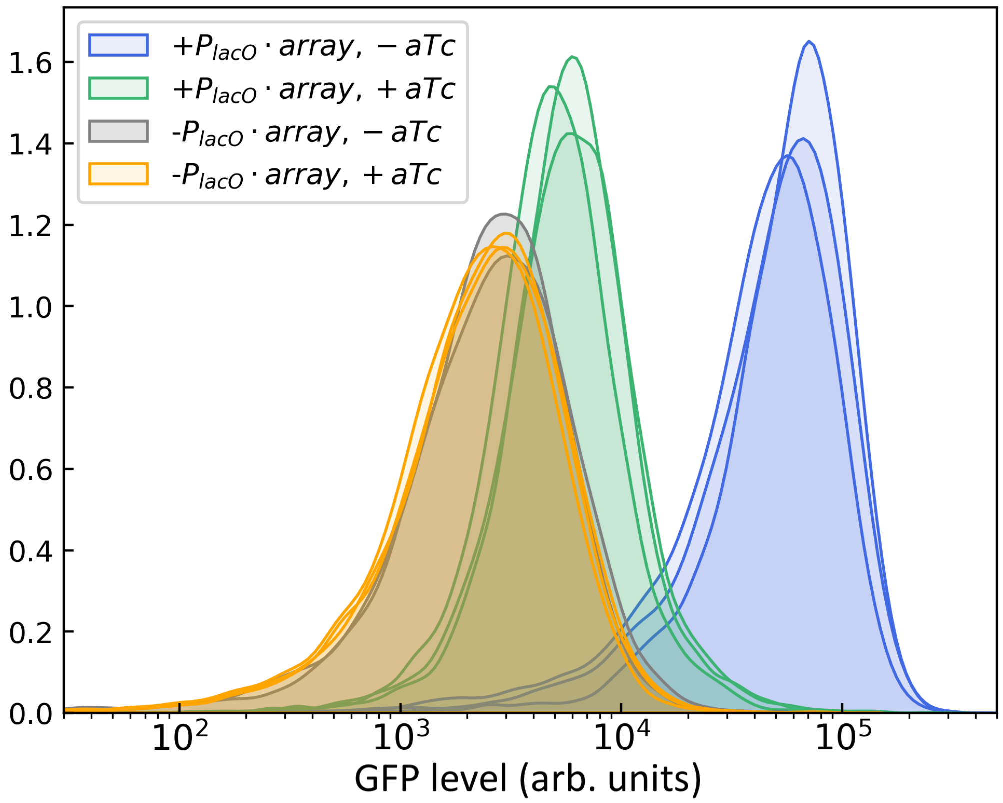

Supplementary Fig. 24: Histogram of GFP fluorescence signals for data in Fig. 2b. Without P_{lacO}-arrays, GFP levels are low regardless of whether aTc is present or not (orange distribution and grey distribution). With P_{lacO}-arrays, the presence of aTc significantly reduces the GFP levels (green distribution versus blue distribution).

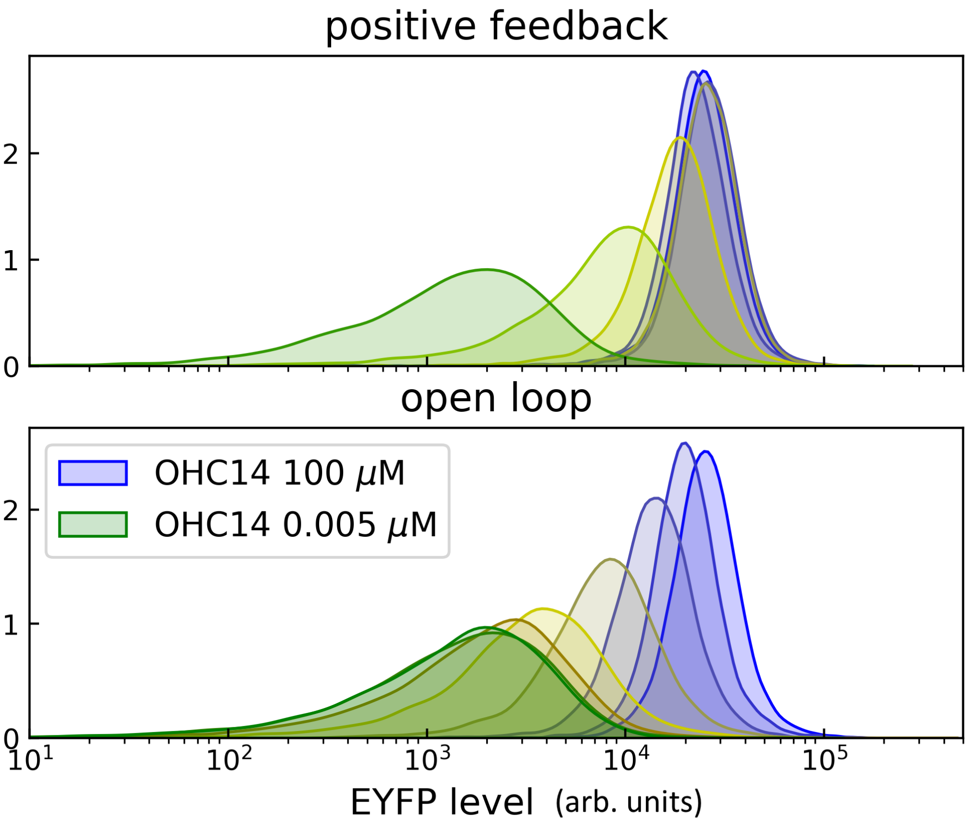

Supplementary Fig. 25: Histograms of EYFP fluorescence signals for data in Fig. 2c. (Upper) Receiver circuit with positive feedback. Histograms measured for one replicate is presented. The histogram of EYFP changes as OHC14 increases from 0.005 μM (green distribution) to 100 μM (blue distribution). (Lower) The same as in the upper panel but for receiver circuit in open loop.

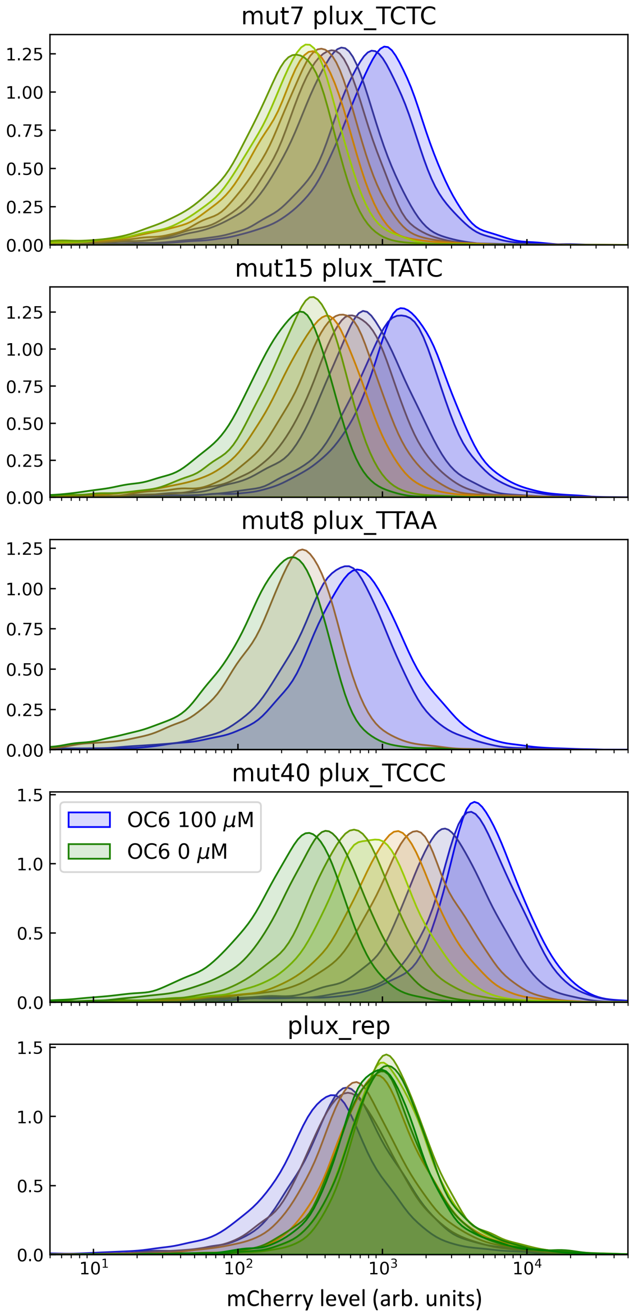

Supplementary Fig. 26: Histograms of mCherry fluorescence signals for data in Fig. 2e. For each P_{lux}, histograms measured for one replicate is presented. The inducer OC6 concentration increases from 0 μM (green distribution) 100 μM (blue distribution).

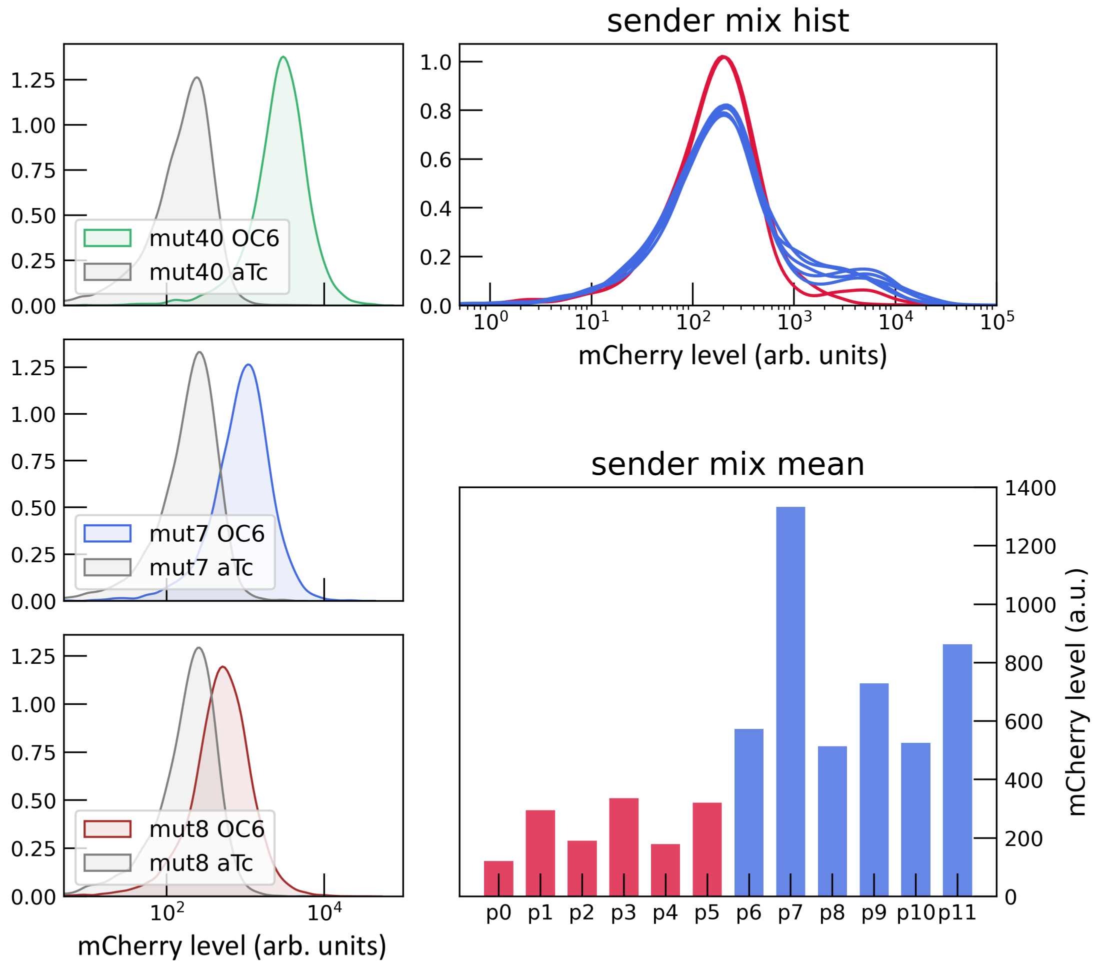

Supplementary Fig. 27: Histograms of mCherry fluorescence measurement for senders involved in one trial of 4-bit pattern experiments as presented in Fig. 3c. (Left) Senders with different P_{lux} promoters incubated either with OC6 or aTc for 210 min. (Right) Histograms of mCherry levels in sender mix (upper) and the mean mCherry values of the sender mix for each pattern (lower).

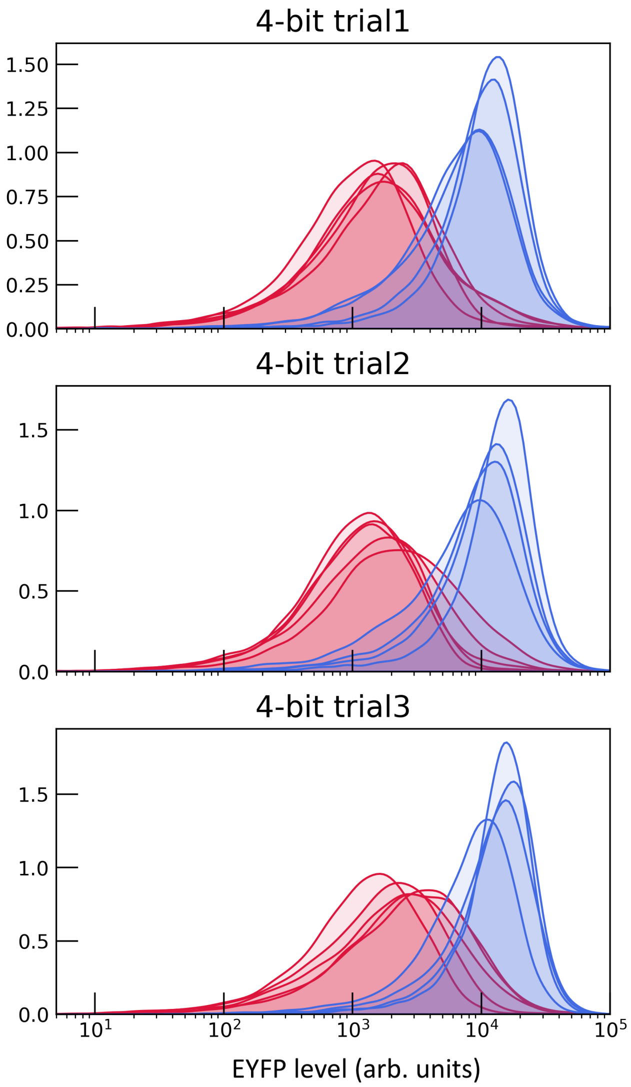

Supplementary Fig. 28: Histograms of EYFP fluorescence levels for receivers in 4-bit patterns as presented in Fig. 3c. Three replicates are presented. Red color represents patterns from 0 to 5, and blue color represents patterns from 6 to 11.

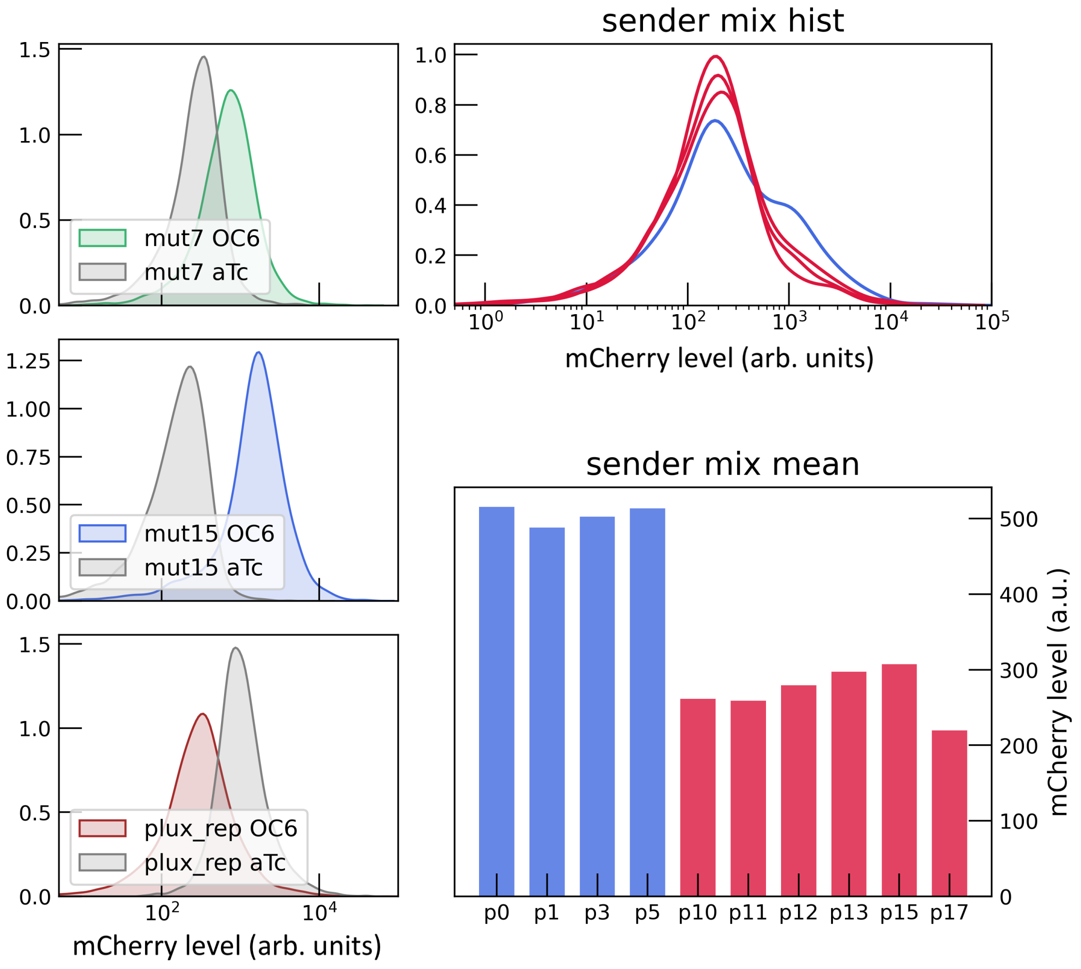

Supplementary Fig. 29: Histograms of mCherry fluorescence measurement for senders involved in one trial of 3×3-bit pattern experiments as presented in Fig. 5c. (Left) Senders with different P_{lux} promoters incubated either with OC6 or aTc for 210 min. (Right) Histograms of mCherry levels in sender mix (upper) and the mean mCherry values of the sender mix for each pattern (lower).

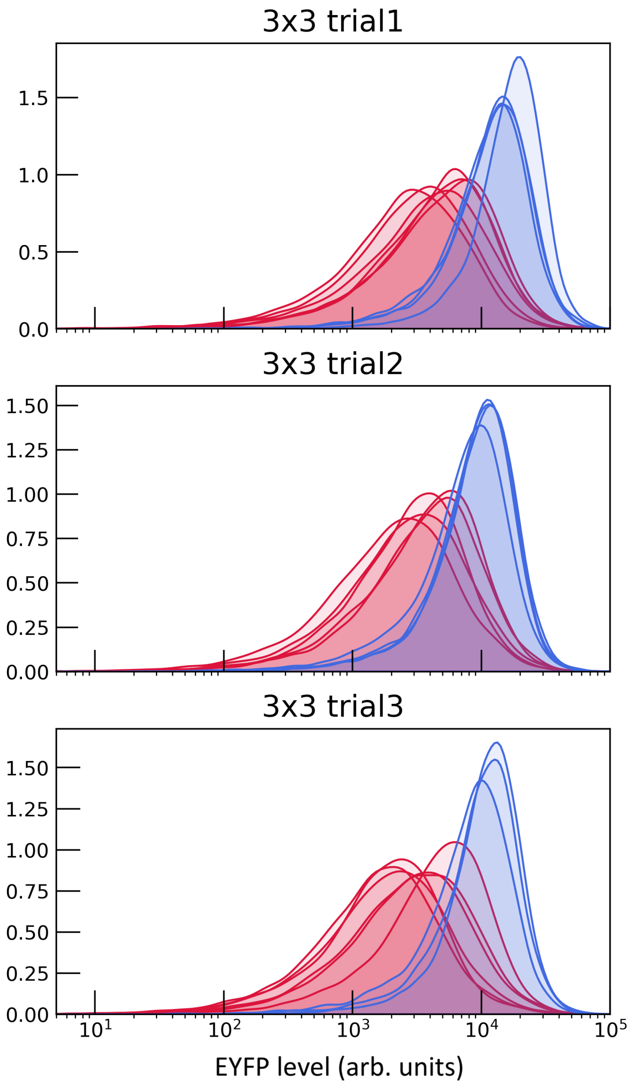

Supplementary Fig. 30: Histograms of EYFP fluorescence levels for receivers in 3×3-bit patterns as presented in Fig. 5c. Three replicates are presented. Red color represents patterns 0, 1, 3 and 5. Blue color represents patterns 10, 11, 12, 13, 15 and 17.

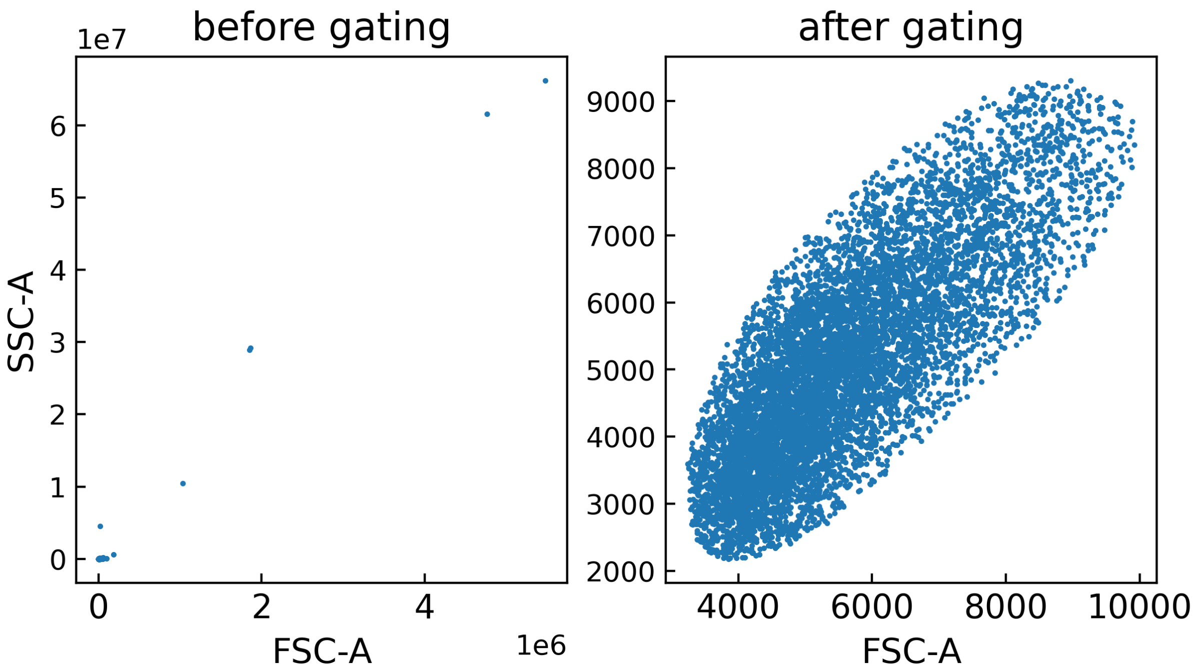

Supplementary Fig. 31: A figure exemplifying the density-based gating strategy on channels 'FSC-A' and 'SSC-A', with a fraction parameter 0.8. The analysis is available from a Python package FlowCal [15].

3 Supplementary videos

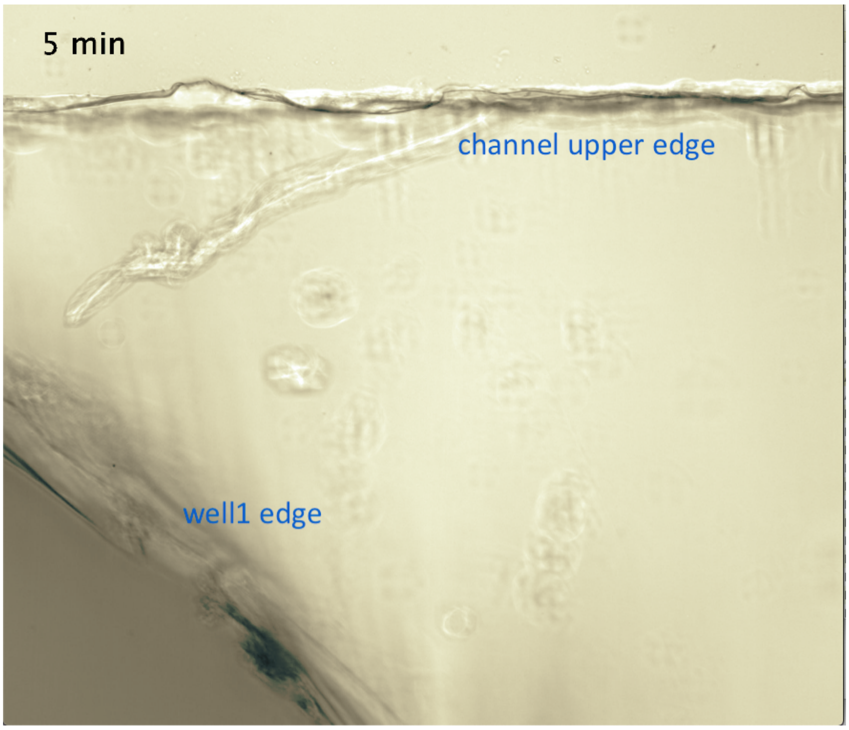

Supplementary Fig. 32: Cell solution was initially added in well1. Cells diffuse rightward during the recording. Recording starts at 5 min after incubation and lasts for 5 hours. Please see uploaded video file SuppVideo1.

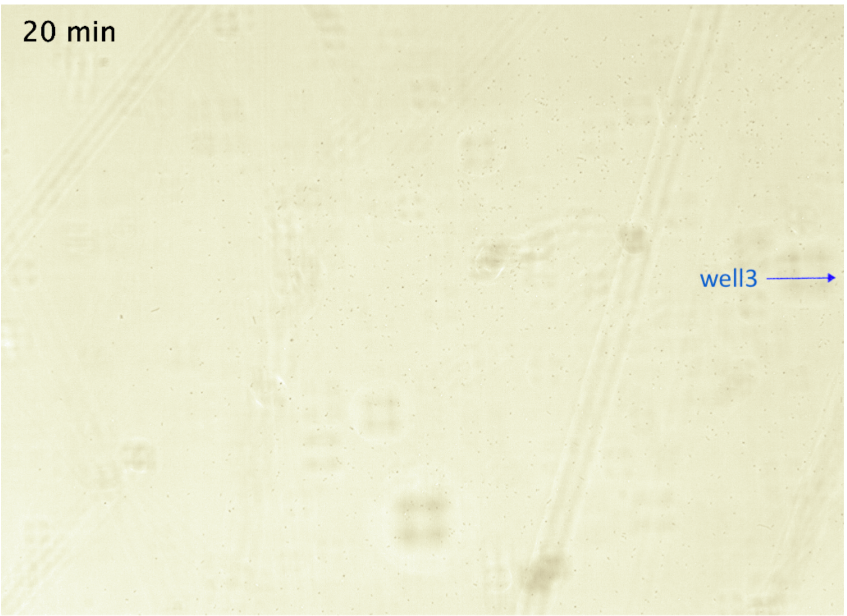

Supplementary Fig. 33: Cell solution was initially added in well3. Cells diffuse leftward during the recording. Some bacteria appear in the channel at the beginning. Recording starts at 20 min after incubation and lasts for more than 5 hours. Please see uploaded video file SuppVideo2.

4 Supplementary tables

Supplementary Table 0. Primers for sequencing plasmids as listed in Fig. S23.

Plasmid	sequencing primer
XL140	cm-f: GGTGGTATATCCAGTGATTTTTTTCTCCAT
	lacI-mid-f: CGGGAAGGCGACTGGAGTGCCATGT
	mCherry-mid-r: CTGGGTCACGGTCACCACGC
	luxR-init-r: TTAATATCATTATTGCTTCTACAAGCTTTAA
XL208	kan-f: GCAAGAAAGCCATCCAGTTTACTTTG
	LCN-r: TCCTGGCATCTTCCAGGAAATCT
XL267	cm-f: GGTGGTATATCCAGTGATTTTTTTCTCCAT
	MCN-r: CAAACTAGCAACACCAGAACAGCCCGTTTG
XL340	kan-f: GCAAGAAAGCCATCCAGTTTACTTTG
	LCN-r: TCCTGGCATCTTCCAGGAAATCT
XL291	cm-f: GGTGGTATATCCAGTGATTTTTTTCTCCAT
	MCN-r: CAAACTAGCAACACCAGAACAGCCCGTTTG
LR191	cm-f: GGTGGTATATCCAGTGATTTTTTTCTCCAT
	MCN-r: CAAACTAGCAACACCAGAACAGCCCGTTTG
LR206	kan-f: GCAAGAAAGCCATCCAGTTTACTTTG
	LCN-r: TCCTGGCATCTTCCAGGAAATCT

Supplementary Table 1. Plasmid combinations for relevant experiments

Plasmids	Figures
XL267+LR206	Fig. 2b
XL267+XL208	Fig. 2b
XL340+XL291	Fig. 2d, Fig. 3c, Fig. 5c
pAJM.1642+LR191	Fig. 2d
XL140mut40+XL208	Fig. 2e, Fig. 3c
XL140mut15+XL208	Fig. 2e, Fig. 5c
XL140mut8+XL208	Fig. 2e, Fig. 3c
XL140mut7+XL208	Fig. 2e, Fig. 3c, Fig. 5c
XL302+XL208	Fig. 2e, Fig. 5c

pAJM.1642 was obtained from Addgene (plasmid #108535).

`

Supplementary Table 2. Crucial promoter and regulatory sequences

prompter names	Sequences
P_{lux}	acctgtaggatcgtacaggtttacgcaagaaaatggtttgttatagtcgaataaa
P_{lux} mut40	tcccgtaggatcgtacaggtttacgcaagaaaatggtttgttatagtcgaataaa
P_{lux} mut15	tatcgtaggatcgtacaggtttacgcaagaaaatggtttgttatagtcgaataaa
P_{lux} mut8	ttaagtaggatcgtacaggtttacgcaagaaaatggtttgttatagtcgaataaa
P_{lux} mut7	tctcgtaggatcgtacaggtttacgcaagaaaatggtttgttatagtcgaataaa
P_{lux} repressor	ttgacggctagctcagtcctaggtacagtgctagcacctgtaggatcgtacaggt
P_{lacO}-array	ttgtgagcggataacaaacgatcgattgtgagcggataacaatgtcctcaggcttgtgagcggataacaacctctatcattgtgagcggataacaatggacgccctgtattgtgagcggataacaacccctgcctttgtgagcggataacaa

-35 box and -10 box are underlined. Operators are bolded. Mutated nucleotides are bolded, italicized and underlined.

Supplementary Table 3 Fitting parameters for transfer functions of senders with PF regulation in Fig. S10a

	K_d (μM)	β_0	β_m	n
Sender PF mut7	7.612E+01	1.372E-02	9.345E+03	0.882
Sender PF mut8	9.999E+02	3.882E-02	4.077E+03	1.513
Sender PF mut40	2.071E-01	1.186E-02	6.575E+03	1.336

Supplementary Table 4 Weights for 3x3-bit patterns (η=20 for all weight vectors)

	Weight values	Corresponding promoters
$\vec{w}_0$	[1813, 1813, -735, -735, 2872, -735, -73<br>5, 1813, 1813]	[m7, m7, nw, nw, m15, nw, nw, m7, m7]

`

$\vec{w}_1$	[1813, -735, 2872, 1813, -735, 1813, -735, 1813, -735]	[m7, nw, m15, m7, nw, m7, nw, m7, nw]
$\vec{w}_2$	[-735, 1813, -735, 1813, -735, 1813, 2872, -735, 1813]	[nw, m7, nw, m7, nw, m7, m15, nw, m7]

"m7", "m8", "m15" represent P_{lux} mut7, P_{lux} mut8 and P_{lux} mut15 respectively. "nw" is short for "negative weights" and represents the P_{lux} repressor.

Supplementary Table 5. Weights for 5x5-bit binary and non-binary patterns (η=20 for all weight vectors)

	Weights for binary patterns	Weights for non-binary patterns
$\vec{w}_0$	[735, 5500, 5500, 735, -735, -735, -735, 735, -735, -735, -735, 735, 5500, 735, -735, -735, -735, 5500, -735, -735, -735, 735, 735, 5500, 735]	[1906, 10000, 2679, 735, 735, 735, 735, 1906, -735, -735, -735, 735, 5500, 735, -735, -735, -735, 10000, -735, 735, -735, 735, 1906, 10000, 2679]
$\vec{w}_1$	[735, -735, -735, 735, 5500, 5500, -735, -735, -735, 5500, 735, 735, -735, 735, 735, -735, 5500, -735, 5500, -735, -735, 735, 735, -735, -735]	[2679, -735, 735, 735, 5500, 5500, -735, -735, -735, 10000, 2679, 735, -735, 735, 735, -735, 10000, -735, 5500, -735, -735, 735, 1906, -735, -735]
$\vec{w}_2$	[-735, -735, -735, 735, -735, -735, 5500, 735, 5500, -735, 735, 735, -735, 735, 735, 5500, -735, -735, -735, 5500, 5500, 735, -735, -735, 735]	[-735, -735, 735, 735, 735, 735, 5500, 1906, 10000, -735, 1906, 735, 735, 735, 735, 10000, -735, -735, 735, 5500, 10000, 735, -735, -735, 2679]

Supplementary Table 6. Weights for 7x7-bit binary and non-binary patterns (η=20 for all weight vectors)

	Weights for binary patterns
$\vec{w}_0$	[1906, 10000, 10000, 10000, 735, 735, 735, 735, 735, 735, 10000, 735, 735, 735, 735, 735, 735, 1906, 735, 735, 735, -735, 735, 735, 10000, 735, 735, -735, 735, 735, 735, 10000, 735, 735, 735, 735, 735, 735, 10000, 735, 735, 735, 735, 735, 735, 1906, 10000, 10000, 1906]
$\vec{w}_1$	[1906, 735, 735, 735, 735, 735, 10000, 10000, 735, 735, 735, 735, 735, 10000, 10000, 735, 735, -735, 735, 735, 10000, 1906, 735, 735, 735, 735, 735, 1906, 735, 10000, 735, 735, 735, 10000, 735, 735, 735, 10000, 735, 10000, 735, 735, 735, 735, 735, 1906, 735, 735, -735]
$\vec{w}_2$	[-735, 735, 735, 735, 735, 735, 735, 735, 735, 735, 735, 735, 735, 735, 735, 10000, 10000, 1906, 10000, 10000, 735, 1906, 735, 735, 735, 735, 735, 1906, 10000, 735, 735, 735, 735, 735, 10000, 10000, 735, 735, 735, 735, 735, 10000, 10000, 735, 735, -735, 735, 735, 1906]

`

	Weights for non-binary patterns
$\vec{w}_0$	[2679, 10000, 5500, 10000, 735, 735, 735, -735, 735, 735, 10000, 735, 735, -735, 735, -735, -73<br>5, 2679, -735, -735, -735, -735, 735, 735, 10000, 735, 735, -735, -735, -735, 735, 10000, 735, -7<br>35, -735, 735, 735, -735, 5500, -735, 735, -735, -735, 735, 735, 2679, 10000, 10000, 1906]
$\vec{w}_1$	[1906, -735, -735, 735, 735, 735, 10000, 10000, 735, 735, -735, 735, 735, 10000, 5500, 735, -73<br>5, -735, 735, -735, 10000, 2679, 735, 735, 735, 735, 735, 735, -735, 10000, 735, -735, 735, 100<br>00, -735, 735, 735, 10000, 735, 10000, 735, -735, 735, 735, 735, 1906, -735, -735, -735]
$\vec{w}_2$	[-735, 735, 735, 735, 735, 735, 735, 735, 735, 735, -735, 735, 735, -735, -735, 10000, 10000, 26<br>79, 10000, 10000, -735, 5500, 735, 735, -735, 735, 735, 735, 10000, -735, 735, -735, 735, -735,<br>10000, 10000, 735, -735, -735, -735, 735, 10000, 10000, 735, 735, -735, -735, 735, 1906]

Supplementary Table 7. Weights for 9x9-bit binary and non-binary patterns (η=20 for all weight vectors)

	Weights for binary patterns
$\vec{w}_0$	[5500, 10000, 10000, 10000, 10000, 735, 735, 735, 735, 735, 735, 735, 735, 10000, 735, 735, 73<br>5, 735, 735, 735, 735, 735, 5500, 735, 735, 735, 735, 735, 735, 735, 735, 10000, 735, 735, 735,<br>735, -735, 735, 735, 735, 10000, 735, 735, 735, -735, 735, 735, 735, 735, 10000, 735, 735, 73<br>5, 735, 735, 735, 735, 735, 10000, 735, 735, 735, 735, 735, 735, 735, 735, 10000, 735, 735, 73<br>5, 735, 735, 735, 735, 735, 5500, 10000, 10000, 10000, 5500]
$\vec{w}_1$	[5500, 735, 735, 735, 735, 735, 735, 735, 10000, 10000, 735, 735, 735, 735, 735, 735, 735, 100<br>00, 10000, 735, 735, 735, -735, 735, 735, 735, 10000, 10000, 735, 735, 735, 735, 735, 735, 735,<br>10000, 5500, 735, 735, 735, 735, 735, 735, 735, 5500, 735, 10000, 735, 735, 735, 735, 735, 10<br>000, 735, 735, 735, 10000, 735, 735, 735, 10000, 735, 735, 735, 735, 735, 10000, 735, 10000, 7<br>35, 735, 735, 735, 735, 735, 735, 5500, 735, 735, 735, -735]
$\vec{w}_2$	[-735, 735, 735, 735, 735, 735, 735, 735, 735, 735, 735, 735, 735, 735, 735, 735, 735, 735, 73<br>5, 735, 10000, 10000, 5500, 10000, 10000, 735, 735, 735, 10000, 735, 735, 735, 735, 735, 1000<br>0, 735, 5500, 735, 735, 735, 735, 735, 735, 735, 5500, 10000, 735, 735, 735, 735, 735, 735, 73<br>5, 10000, 10000, 735, 735, 735, 735, 735, 735, 735, 10000, 10000, 735, 735, 735, 735, 735, 73<br>5, 735, 10000, 10000, 735, 735, 735, -735, 735, 735, 735, 5500]
	**Weights for non-binary patterns**
$\vec{w}_0$	[2679, 10000, 10000, 10000, 10000, 735, 735, 735, -735, -735, 735, 735, 735, 10000, 735, 735, 735, -735,<br>-735, 735, -735, -735, 2679, -735, -735, 735, -735, -735, -735, 735, 735, 10000, 735, 735, -735, -735,<br>-735, 735, 735, 735, 10000, 735, 735, 735, -735, -735, -735, 735, 735, 10000, 735, 735, -735, -735, -735,<br>735, -735, 735, 10000, 735, -735, 735, -735, -735, 735, 735, -735, 10000, -735, 735, 735, 735, 735, 735,<br>735, 735, 5500, 10000, 10000, 10000, 5500]
$\vec{w}_1$	[5500, -735, -735, -735, -735, 735, 735, 735, 10000, 10000, 735, 735, 735, -735, 735, 735, 735, 10000,<br>10000, 735, -735, -735, -735, -735, -735, 735, 10000, 10000, -735, 735, 735, -735, 735, 735, -735, 10000,<br>5500, 735, 735, 735, -735, 735, 735, 735, 1906, -735, 10000, 735, 735, -735, 735, 735, 10000, -735, -735,<br>735, 10000, 735, -735, 735, 10000, 735, -735, -735, 735, 735, 10000, -735, 10000, 735, 735, -735, -735,<br>735, 735, 735, 5500, -735, -735, -735, -735]
$\vec{w}_2$	[-735, -735, 735, -735, -735, 735, 735, 735, -735, -735, 735, 735, 735, -735, 735, 735, 735, -735, -735,<br>735, 10000, 10000, 2679, 10000, 10000, 735, -735, 735, 10000, 735, 735, -735, 735, 735, 10000, -735,<br>5500, 735, 735, 735, -735, 735, 735, 735, 1906, 10000, -735, 735, 735, -735, 735, 735, -735, 10000,<br>10000, 735, -735, 735, -735, 735, -735, 735, 10000, 10000, 735, 735, -735, -735, -735, 735, 735, 10000,<br>10000, 735, 735, 735, -735, -735, -735, -735, 5500]

`

References

1. Weber, M. & Buceta, J. Noise regulation by quorum sensing in low mRNA copy number systems. *BMC Systems Biology* **5,** 11. ISSN: 1752-0509 (Dec. 2011).

2. Hooshangi, S., Thiberge, S. & Weiss, R. Ultrasensitivity and noise propagation in a synthetic transcriptional cascade. *Proceedings of the National Academy of Sciences* **102,** 3581–3586. ISSN: 0027-8424, 1091-6490 (Mar. 2005).

3. Bishop, C. *Pattern recognition and machine learning* ISBN: 9780387310732 (Springer, 2006).

4. Kochenderfer, M. J. & Wheeler, T. A. *Algorithms for optimization* ISBN: 9780262039420 (The MIT Press, 2019).

5. Baldassi, C., Braunstein, A., Brunel, N. & Zecchina, R. Efficient supervised learning in networks with binary synapses. *Proceedings of the National Academy of Sciences* **104,** 11079–11084. ISSN: 0027-8424, 1091-6490 (June 2007).

6. Saltelli, A. *et al.* Variance based sensitivity analysis of model output. Design and estimator for the total sensitivity index. *Computer Physics Communications* **181,** 259–270. ISSN: 00104655 (Feb. 2010).

7. Herman, J. & Usher, W. SALib: An open-source Python library for Sensitivity Analysis. *The Journal of Open Source Software* **2,** 97. ISSN: 2475-9066 (Jan. 2017).

8. Fetzner, S. Quorum quenching enzymes. *Journal of Biotechnology* **201,** 2–14. ISSN: 01681656 (May 2015).

9. MacKay, D. J. C. *Information theory, inference, and learning algorithms* ISBN: 9780521642989 (Cambridge University Press, 2003).

10. Gardner, E. Maximum Storage Capacity in Neural Networks. *Europhysics Letters (EPL)* **4,** 481–485. ISSN: 0295-5075, 1286-4854 (Aug. 1987).

11. Krauth, W. & Mézard, M. Storage capacity of memory networks with binary couplings. *Journal de Physique* **50,** 3057–3066. ISSN: 0302-0738 (1989).

12. Tamsir, A., Tabor, J. J. & Voigt, C. A. Robust multicellular computing using genetically encoded NOR gates and chemical 'wires'. *Nature* **469,** 212–215 (Jan. 2011).

13. Kylilis, N., Tuza, Z. A., Stan, G.-B. & Polizzi, K. M. Tools for engineering coordinated system behaviour in synthetic microbial consortia. *Nature Communications* **9,** 2677. ISSN: 2041-1723 (Dec. 2018).

14. Lambert, T. J. FPbase: a community-editable fluorescent protein database. *Nature Methods* **16,** 277–278. ISSN: 1548-7091, 1548-7105 (Apr. 2019).

15. Castillo-Hair, S. M. *et al.* FlowCal: A User-Friendly, Open Source Software Tool for Automatically Converting Flow Cytometry Data from Arbitrary to Calibrated Units. *ACS Synthetic Biology* **5,** 774–780. ISSN: 2161-5063, 2161-5063 (July 2016).
