# Supplementary material for: Synthetic neural-like computing in microbial consortia for pattern recognition
Source: Nat Commun. 2021 May 25;12:3139. doi: 10.1038/s41467-021-23336-0 (PMC8149857; doi:10.1038/s41467-021-23336-0)
Supplement: Supplementary file 5 — Reporting Summary [file 41467_2021_23336_MOESM5_ESM.pdf]

nature research

Corresponding author(s): Ramez Daniel

Last updated by author(s): 04-18-2021

Reporting Summary

Nature Research wishes to improve the reproducibility of the work that we publish. This form provides structure for consistency and transparency in reporting. For further information on Nature Research policies, see our Editorial Policies and the Editorial Policy Checklist.

Statistics

For all statistical analyses, confirm that the following items are present in the figure legend, table legend, main text, or Methods section.

n/a	Confirmed	
☐	☒	The exact sample size (n) for each experimental group/condition, given as a discrete number and unit of measurement
☐	☒	A statement on whether measurements were taken from distinct samples or whether the same sample was measured repeatedly
☒	☐	The statistical test(s) used AND whether they are one- or two-sided<br>*Only common tests should be described solely by name; describe more complex techniques in the Methods section.*
☒	☐	A description of all covariates tested
☒	☐	A description of any assumptions or corrections, such as tests of normality and adjustment for multiple comparisons
☐	☒	A full description of the statistical parameters including central tendency (e.g. means) or other basic estimates (e.g. regression coefficient) AND variation (e.g. standard deviation) or associated estimates of uncertainty (e.g. confidence intervals)
☒	☐	For null hypothesis testing, the test statistic (e.g. F, t, r) with confidence intervals, effect sizes, degrees of freedom and P value noted<br>*Give P values as exact values whenever suitable.*
☒	☐	For Bayesian analysis, information on the choice of priors and Markov chain Monte Carlo settings
☒	☐	For hierarchical and complex designs, identification of the appropriate level for tests and full reporting of outcomes
☒	☐	Estimates of effect sizes (e.g. Cohen's d, Pearson's r), indicating how they were calculated

Our web collection on statistics for biologists contains articles on many of the points above.

Software and code

Policy information about availability of computer code

Data collection	We used DNAplotlib (v1.0) to generate circuit diagrams and used FlowCal (v1.2.2) to analyze FACS data.
Data analysis	The algorithm code for weight optimization and experiment data analysis are available on GitHub with DOI [10.5281/zenodo.4682962].

For manuscripts utilizing custom algorithms or software that are central to the research but not yet described in published literature, software must be made available to editors and reviewers. We strongly encourage code deposition in a community repository (e.g. GitHub). See the Nature Research guidelines for submitting code & software for further information.

Data

Policy information about availability of data

All manuscripts must include a data availability statement. This statement should provide the following information, where applicable:

- Accession codes, unique identifiers, or web links for publicly available datasets
- A list of figures that have associated raw data
- A description of any restrictions on data availability

The FACS experiment data underlying Fig. 2b, 2d, 2e, 3c, 4c and 5c have been deposited to flowrepository with IDs FR-FCM-Z3CK [https://flowrepository.org/id/ FR-FCM-Z3CK], FR-FCM-Z3CW [https://flowrepository.org/id/FR-FCM-Z3CW], FR- FCM-Z3D6 [https://flowrepository.org/id/FR-FCM-Z3D6], FR-FCM-Z3DG [https: // flowrepository.org/id/FR-FCM-Z3DG], FR-FCM-Z3MQ [https://flowrepository. org/id/FR-FCM-Z3MQ], and FR-FCM-Z3DD [https://flowrepository.org/id/FR-FCM-Z3DD]. These data are also available in GitHub with DOI [10.5281/zenodo.4682962] under 'experiment_data' subdirectory. Plasmids with maps in Supplementary Fig. 23 are included in the same GitHub repository. Source data are provided with this paper. All other relevant data are available from the authors upon request.

Field-specific reporting

Please select the one below that is the best fit for your research. If you are not sure, read the appropriate sections before making your selection.

[x] Life sciences [ ] Behavioural & social sciences [ ] Ecological, evolutionary & environmental sciences

For a reference copy of the document with all sections, see nature.com/documents/nr-reporting-summary-flat.pdf

Life sciences study design

All studies must disclose on these points even when the disclosure is negative.

Sample size	In most experiments presented in the main text, we collected from three independent samples. In Fig.2e, we repeated more than three samples as indicated in the figure caption in order to characterize circuit transfer functions from their inherent noise.
Data exclusions	No data was excluded from the analyses.
Replication	Performed three independent replicates. The results are highly reproducible across replicates. In Fig.2e, more than three replicates were performed.
Randomization	Sample colonies were randomly selected and incubated overnight for experiments.
Blinding	All samples used in this work were prepared by the investigators in the laboratory, together with appropriate controls. Therefore, blinding of the investigators is not relevant to our study

Reporting for specific materials, systems and methods

We require information from authors about some types of materials, experimental systems and methods used in many studies. Here, indicate whether each material, system or method listed is relevant to your study. If you are not sure if a list item applies to your research, read the appropriate section before selecting a response.

Materials & experimental systems

n/a	Involved in the study	
[x]	[ ]	Antibodies
[x]	[ ]	Eukaryotic cell lines
[x]	[ ]	Palaeontology and archaeology
[x]	[ ]	Animals and other organisms
[x]	[ ]	Human research participants
[x]	[ ]	Clinical data
[x]	[ ]	Dual use research of concern

Methods

n/a	Involved in the study	
[x]	[ ]	ChIP-seq
[ ]	[x]	Flow cytometry
[x]	[ ]	MRI-based neuroimaging

Flow Cytometry

Plots

Confirm that:

[x] The axis labels state the marker and fluorochrome used (e.g. CD4-FITC).

[x] The axis scales are clearly visible. Include numbers along axes only for bottom left plot of group (a 'group' is an analysis of identical markers).

[x] All plots are contour plots with outliers or pseudocolor plots.

[x] A numerical value for number of cells or percentage (with statistics) is provided.

Methodology

Sample preparation	E. coli cell culture incubated for various period, with or without inducers.
Instrument	Beckman Coulter CytoFlex
Software	Python FlowCal (v1.2.2) open source software
Cell population abundance	10000 events without gating

Gating strategy

Density based gating to gate FSC-A and SSC-A, with a fraction of 0.8, as shown in Fig.S31

☒ Tick this box to confirm that a figure exemplifying the gating strategy is provided in the Supplementary Information.
