# Supplementary material for: Synthetic neural-like computing in microbial consortia for pattern recognition
Source: Nat Commun. 2021 May 25;12:3139. doi: 10.1038/s41467-021-23336-0 (PMC8149857; doi:10.1038/s41467-021-23336-0)
Supplement: Supplementary file 6 — Description of Additional Supplementary Files [file 41467_2021_23336_MOESM6_ESM.pdf]

Title: Supplementary Movie 1

Description: Cell solution was initially added in well1. Cells diffuse rightward during the recording. Recording starts at 5 min after incubation and lasts for 5 hours. Please see uploaded video file SuppVideo1.

Title: Supplementary Movie2

Description: Cell solution was initially added in well3. Cells diffuse leftward during the recording. Some bacteria appear in the channel at the beginning. Recording starts at 20 min after incubation and lasts for more than 5 hours. Please see uploaded video file SuppVideo2.
